# Guanosine Tetraphosphate Has a Similar Affinity for Each of Its Two Binding Sites on *Escherichia coli* RNA Polymerase

**DOI:** 10.3389/fmicb.2020.587098

**Published:** 2020-11-05

**Authors:** Angela R. Myers, Danielle P. Thistle, Wilma Ross, Richard L. Gourse

**Affiliations:** Department of Bacteriology, University of Wisconsin-Madison, Madison, WI, United States

**Keywords:** bacterial transcription, RNA polymerase, ppGpp, DksA, omega subunit, stringent response

## Abstract

During nutrient deprivation, the bacterial cell undergoes a stress response known as the stringent response. This response is characterized by induction of the nucleotide derivative guanosine tetraphosphate (ppGpp) that dramatically modulates the cell’s transcriptome. In *Escherichia coli*, ppGpp regulates transcription of as many as 750 genes within 5 min of induction by binding directly to RNA polymerase (RNAP) at two sites ~60 Å apart. One proposal for the presence of two sites is that they have different affinities for ppGpp, expanding the dynamic range over which ppGpp acts. We show here, primarily using the Differential Radial Capillary Action of Ligand Assay (DRaCALA), that ppGpp has a similar affinity for each site, contradicting the proposal. Because the ppGpp binding sites are formed by interactions of the β’ subunit of RNAP with two small protein factors, the ω subunit of RNAP which contributes to Site 1 and the transcription factor DksA which contributes to Site 2, variation in the concentrations of ω or DksA potentially could differentially regulate ppGpp occupancy of the two sites. It was shown previously that DksA varies little at different growth rates or growth phases, but little is known about variation of the ω concentration. Therefore, we raised an anti-ω antibody and performed Western blots at different times in growth and during a stringent response. We show here that ω, like DksA, changes little with growth conditions. Together, our data suggest that the two ppGpp binding sites fill in parallel, and occupancy with changing nutritional conditions is determined by variation in the ppGpp concentration, not by variation in ω or DksA.

## Introduction

When nutritional resources change, cells adjust their transcriptional output to match the new environment. In almost all bacterial species, this is accomplished in part by synthesis of the secondary messengers guanosine tetraphosphate (ppGpp; guanosine 5'-diphosphate 3'-diphosphate) and guanosine pentaphosphate (pppGpp; guanosine 5'-triphosphate 3'-diphosphate), respectively, collectively referred to here as ppGpp (reviewed in Hauryliuk et al., 2015; Liu et al., 2015; Irving and Corrigan, 2018). In proteobacteria like *Escherichia coli*, the basal level of ppGpp (from ~1 to 10 μM) only moderately affects gene expression, but induction of RelA in response to the accumulation of deacylated tRNA(s) increases the ppGpp concentration 100–1,000-fold, dramatically changing gene expression (Ryals et al., 1982; Varik et al., 2017).

In this so-called stringent response, transcription of hundreds of genes, many of which are related to translation, is inhibited within 5 min of ppGpp induction, and transcription of hundreds of other genes, many of which are related to pathways involved in amino acid biosynthesis, is stimulated (Durfee et al., 2008; Traxler et al., 2008; Sanchez-Vazquez et al., 2019). This reprogramming of the transcriptome is accomplished in *E. coli* by direct binding of ppGpp to RNA polymerase (RNAP; Sanchez-Vazquez et al., 2019). ppGpp also binds directly to many proteins other than RNAP, altering their activities and contributing further to the remodeling of cellular metabolism (Zhang et al., 2018; Wang et al., 2019).

**Figure 1:**
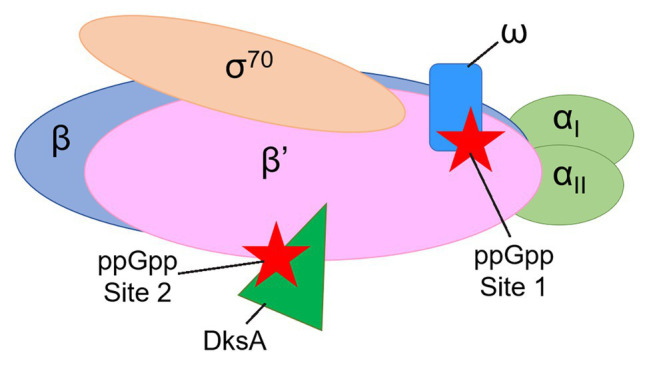
Cartoon representation of *Escherichia coli* RNA polymerase (RNAP) showing the locations of guanosine tetraphosphate (ppGpp) binding Sites 1 and 2. The αI and αII subunits (a homodimer, encoded by the *rpoA* gene) are shown in light green. The β subunit (encoded by the *rpoB* gene) is in blue. The β’ subunit (encoded by the *rpoC* gene) is in pink. The ω subunit (encoded by the *rpoZ* gene) is in blue. The σ^70^ subunit (encoded by the *rpoD* gene) is in tan. ppGpp (red stars) binds at the interface of the ω and β’ subunits (Site 1) and at the interface of the transcription factor DksA (dark green) and the β’ subunit (Site 2).

Two proteins that are not essential for the catalytic activity of *E. coli* RNAP are nevertheless required for the effects of ppGpp on transcription initiation, the 10.2 kDa RNAP subunit ω and the 17.5 kDa transcription factor DksA (Paul et al., 2004, 2005; Vrentas et al., 2005). Genetic and biochemical evidence indicated that ppGpp binds to two sites on RNAP ~60 Å apart (Ross et al., 2013, 2016), with Site 1 at the interface of the ω and β’ subunits of RNAP and Site 2 at the interface of DksA and the secondary channel rim of the β’ subunit (Figure 1). Crystal structures of the RNAP-ppGpp complex are consistent with the models based on the genetic and biochemical studies, and indicate that at Site 1 the ppGpp phosphates are coordinated by residues 2–5 and other residues in ω, as well as by residues R417, K615 in β’. The guanine base is coordinated by several residues in β’, including I619, D622, and R362 (Mechold et al., 2013; Zuo et al., 2013). A strain lacking *rpoZ*, the gene encoding the RNAP ω subunit, (i.e., lacking Site 1) displays a modest lag in recovering from a downshift from a rich to a minimal medium (Gentry et al., 1991; Ross et al., 2013, 2016), while strains lacking *dksA* have more pronounced defects in recovery from a downshift and in transcriptional regulation by ppGpp (Paul et al., 2004; Ross et al., 2016; Sanchez-Vazquez et al., 2019).

The two ppGpp binding sites in RNAP are generally conserved among proteobacteria based on conservation of the residues that contribute to binding (Ross et al., 2013, 2016). However, where it has been investigated, the effects of ppGpp on transcription in some evolutionarily distant bacterial phyla do not involve direct binding of ppGpp to RNAP. For example, in *Bacillus subtilis*, ppGpp inhibits transcription by binding to protein targets involved in nucleotide metabolism, leading to reduced levels of GTP, the initiating nucleotide for many promoters (Krasny and Gourse, 2004; Liu et al., 2015).

The identification of a second ppGpp-binding pocket (Site 2) in *E. coli* RNAP provided an explanation for why disruption of Site 1 had only a modest effect on the stringent response. Analysis of the effects of specific mutations in *dksA* or *rpoC* (the gene encoding β’) on transcription *in vitro*, crosslinking of 6-thio-ppGpp and binding of ppGpp to the RNAP-DksA complex suggested that Site 2 is at the interface of DksA and the β’ subunit rim helices at the entrance to the RNAP secondary channel (Ross et al., 2016). These results and a subsequent crystal structure of an RNAP-DksA-ppGpp complex indicated that residues defined by the mutational studies, including K98, R91, and K139 in DksA, coordinate the phosphates of ppGpp (Molodtsov et al., 2018), and the guanosine base is coordinated by two additional residues implicated by the mutational studies, β’ N680 and DksA L95 (Ross et al., 2016; Molodtsov et al., 2018). A recent cryo-EM structure, in which flexible regions of RNAP were not constrained by crystal packing forces, showed that DksA R129 is also in direct contact with ppGpp (Chen, unpublished), consistent with biochemical analysis of effects of DksA substitution variants on ppGpp binding and function at Site 2 (Ross et al., 2016).

Guanosine tetraphosphate binding to Site 1 alone (in the absence of DksA) has a modest inhibitory effect on transcription from the *rrnB* P1 promoter at saturating ω concentrations (~3-fold), and does not activate transcription from amino acid biosynthesis promoters. ppGpp binding to Site 2 alone (i.e., with a near saturating concentration of DksA but in the absence of the ω subunit) has a larger inhibitory effect on *rrnB* P1 (~6-fold) and is sufficient for full activation of amino acid biosynthesis promoters (Vrentas et al., 2005; Ross et al., 2016). When both ppGpp binding sites are present, inhibition of *rrnB* P1 is greater than with either site alone (15–20-fold). These results are consistent with the growth properties of strains lacking ppGpp (Xiao et al., 1991), ω (Gentry and Burgess, 1989; Gentry et al., 1991), or DksA (Paul et al., 2004, 2005), or containing only Site 1 (Ross et al., 2013), only Site 2 (Ross et al., 2016), or both (Ross et al., 2016; Sanchez-Vazquez et al., 2019).

The evolutionary rationale for having two sites is unclear. One model is that the two sites have different affinities for ppGpp, expanding the dynamic range over which ppGpp acts. To test that model, here, we use the Differential Radial Capillary Action of Ligand Assay (DRaCALA; Roelofs et al., 2011), as modified to measure ppGpp binding to RNAP (Ross et al., 2016), to determine the binding affinities of ppGpp for each of the two sites on *E. coli* RNAP independently as well as together.

We find that both binding sites have similar intrinsic affinities for ppGpp. In addition, we find that the concentrations of ω at different times in cell growth vary only slightly. In conjunction with previous measurements of the concentrations of DksA *in vivo* (Rutherford et al., 2007), our results indicate that the two binding sites fill with ppGpp in parallel and not sequentially as ppGpp concentrations increase. Furthermore, the binding affinities of RNAP for ppGpp that we determined *in vitro* are consistent with the reported effects of ppGpp on transcription even in non-stressed conditions *in vivo*.

## Materials and Methods

### Strains and Plasmids

Strains and plasmids are listed in Supplementary Table S1 in Supplemental data. To construct the pET23a-His10-SUMO-*rpoZ* plasmid used to purify ω for antibody development, a gene block (Integrated DNA Technologies) of *rpoZ* was inserted into pET23a-His10-SUMO (Invitrogen; RLG14235) at the BamH1 and HindIII sites using HiFi DNA Assembly (NEB) to create RLG15371.

### Purification of Proteins

Purification of RNAP [wild-type (WT) or mutant; Supplementary Figure S1], the ω subunit of RNAP (Supplementary Figure S2), DksA (WT and variants), GreB, and TraR were as described in Expanded Materials and Methods.

### Measuring Binding Affinities by DRaCALA

Binding of [^32^P]-ppGpp to RNAP, RNAP/DksA, RNAP/TraR, or RNAP/GreB complexes was measured by the DRaCALA, adapted from Roelofs et al. (2011). See Expanded Materials and Methods for details.

### Mathematical Modeling of ppGpp Binding

The most widely used mathematical model for multisite ligand binding to a protein was first proposed by Hill (1910). By plotting fractional binding of the enzyme as a function of ligand concentration, one can calculate the dissociation constant and the “Hill coefficient,” which indicates whether the binding of multiple ligands is positively or negatively cooperative (see Expanded Materials and Methods for details).

### Statistical Analysis

All K_d,app_ values for each set of binding experiments were subjected to a rank sum test (Sigma Plot) to determine if the K_d,app_ values in each set were statistically different from other values. Statistically different values were then given a *p*-value.

### Western Blots

Polyclonal antibodies were raised by Covance, Inc., following injection of rabbits with purified ω. Quantitative western blots were performed on cells grown in LB as described in Expanded Materials and Methods.

## Results

### ppGpp Binds With a Similar Affinity to Sites 1 and 2 on RNAP

**Figure 2:**
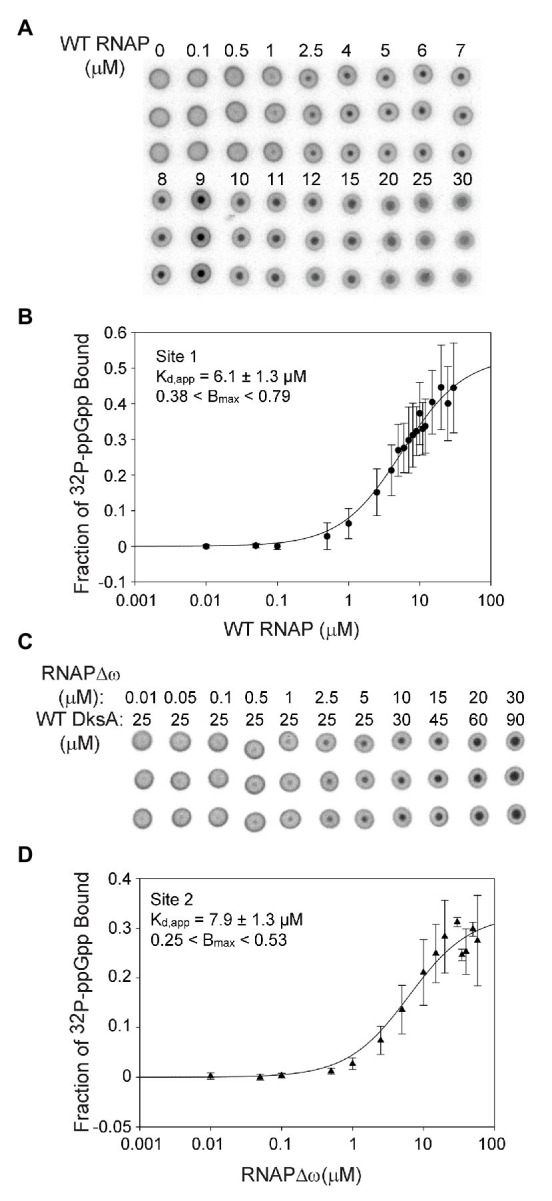
Sites 1 and 2 have similar binding affinities for ppGpp. **(A)** Differential Radial Capillary Action of Ligand Assay (DRaCALA) analysis of [^32^P]-ppGpp binding to Site 1. Increasing amounts of wild-type (WT) RNAP (without DksA) were equilibrated with a constant amount of [^32^P]-ppGpp in DRaCALA buffer and spotted on nitrocellulose filters. Triplicate filters from one representative experiment are shown. **(B)** The plot shown is a one-site saturation binding curve using averaged [^32^P]-ppGpp binding data from seven experiments conducted with five separate preparations of wild-type RNAP. K_d,app_ and B_max_ values were determined by fitting each individual experiment to a one-site saturation ligand binding curve. K_d,app_ and B_max_ values and error shown in the inset were averaged from the values for the seven experiments. See Expanded Materials and Methods for further details. **(C)** Representative DRaCALA analysis of [^32^P]-ppGpp binding to Site 2. Same as in **(A)** except RNAP lacked the ω subunit (RNAPΔω), and DksA was included. **(D)** Same as **(B)** except the reaction contained RNAPΔω and DksA.

**Figure 3:**
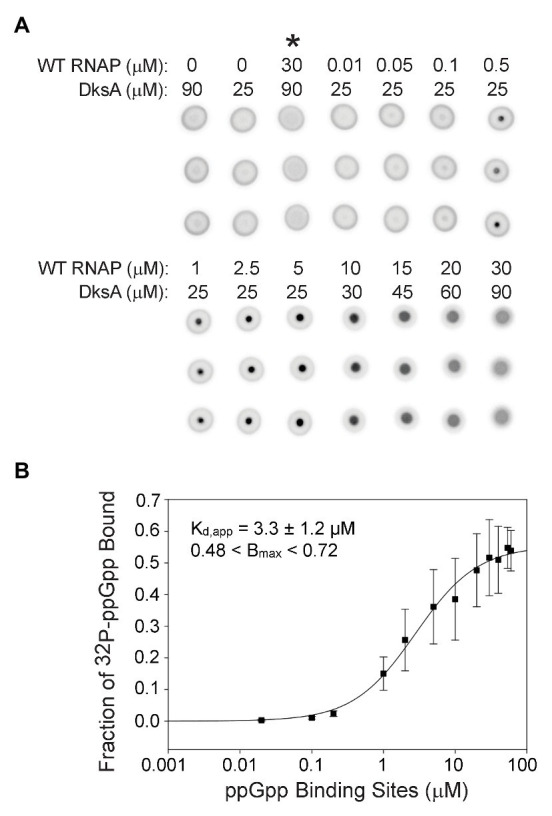
Guanosine tetraphosphate binds with a higher affinity when RNAP contains both ppGpp binding sites. **(A)** [^32^P]-ppGpp binding to wild-type RNAP in the presence of DksA as measured by DRaCALA. Triplicate filters from one representative experiment are shown. Wild-type RNAP and DksA concentrations are indicated above each column of three filters. Control reactions are in the first three columns. Asterisk above the third column indicates the presence of unlabeled competitor ppGpp (1 mM) in the reaction. **(B)** The plot shown is a one-site saturation binding curve for the average [^32^P]-ppGpp binding data with the error bars representing one SD from the mean from seven independent experiments conducted with five individually purified preparations of WT RNAP, as described in the legend for Figure 2. The K_d,app_, B_max_, and error shown in the inset were determined by averaging K_d,app_ values from each of the individual experiments, as in Figure 2.

Our previous studies evaluated the relative roles of Sites 1 and 2 on the transcriptional effects of ppGpp, an indirect indicator of ppGpp binding to RNAP (Ross et al., 2013, 2016). These experiments showed that Site 2 had a much larger effect on both inhibition and activation than Site 1, even though the concentration of ppGpp needed for half-maximal effects on transcription appeared similar, ~12–21 μM for Site 1 at saturating ω and ~19 μM for Site 2 at nearly saturating DksA (Ross et al., 2013, 2016). To measure ppGpp binding more directly, we used DRaCALA (Roelofs et al., 2011; Ross et al., 2016) to determine the affinities of ppGpp for each of the two sites on RNAP independently (Figure 2) as well as together (Figure 3). The specificity of this assay for ppGpp was established previously using unlabeled nucleotides as competitors (Supplementary Figure S3A; Ross et al., 2016). Unlabeled ppGpp competed for binding of ^32^P-ppGpp to each of the two sites, while GDP partially competed, and ATP did not compete at all. As this is a non-equilibrium binding assay, the K_d_ values are reported here as apparent K_d_ (K_d,app_) values. A table summarizing all the apparent K_d_ values reported here is provided as Supplementary Table S2.

For measuring binding of ppGpp to Site 1, wild-type RNAP was purified by concurrent overproduction of the four subunits of core RNAP, α, β, and β’ encoded by one plasmid and overproduction of ω encoded by a second plasmid (see Expanded Materials and Methods). DksA was not included in the binding reactions with wild-type RNAP and ppGpp, eliminating binding to Site 2 (Ross et al., 2016). Increasing amounts of RNAP were combined with a fixed low concentration of [^32^P]-ppGpp (~5 nM). The observed fraction of [^32^P]-ppGpp bound at each RNAP concentration from each of seven independent experiments was fit to a one site saturation binding curve (see Expanded Materials and Methods for description of curve fitting). The K_d,app_’s for each independent binding curve were then averaged, resulting in a K_d,app_ of 6.1 ± 1.3 μM for ppGpp binding to Site 1 (Figures 2A,B).

We ensured that the ω subunit was saturating in the RNAP preparations by adding increasing amounts of purified ω to the binding reaction and measuring the fraction of [^32^P]-ppGpp bound (Supplementary Figure S3B). The additional ω did not increase binding of ppGpp, indicating that ω was already saturating in our RNAP preparation derived from cells overexpressing ω. In contrast, we showed previously that effects of ppGpp on transcription increased slightly when RNAP was purified without concurrent overproduction of ω (Vrentas et al., 2005).

The Site 2 ppGpp binding pocket consists of residues from both DksA and β’ (Ross et al., 2016; Molodtsov et al., 2018). For measuring binding of [^32^P]-ppGpp to Site 2, independent of ppGpp binding to Site 1, we used logic similar to that described above, but with purified DksA added to the reactions and using an RNAP lacking ω (RNAPΔω) obtained by purification of RNAP from a strain deleted for *rpoZ* (*ΔrpoZ*). We showed previously that [^32^P]-ppGpp does not bind to DksA in the absence of RNAP (Ross et al., 2016), and as described above for Site 1, unlabeled ppGpp competed with [^32^P]-ppGpp for binding to Site 2 (Supplementary Figure S3A). The data from each of seven separate experiments measuring ppGpp binding to Site 2 were fit to one-site saturation binding curves, and the K_d,app_ values from each experiment were averaged (Figures 2C,D). The K_d,app_ for Site 2 was 7.9 ± 1.3 μM, similar to the ppGpp binding affinity for Site 1.

### ppGpp Binds With Very low Affinity to RNAP Lacking Sites 1 and 2

For comparison with the affinities measured above, we measured binding of [^32^P]-ppGpp to RNAPs that we had shown previously by *in vitro* transcription did not respond to ppGpp. The K_d,app_ for an RNAP purified from a strain without ω (Δ*rpoZ*) to which no DksA was added (and thus lacked Sites 1 and 2) was >100 μM (Supplementary Figure S4A). Similarly, RNAP purified from a strain without ω (Δ*rpoZ*) overexpressing ωΔ2–5 (RNAPΔω + ωΔ2–5) also had a K_d,app_ >100 μM (Supplementary Figure S4B). We define the very weak binding of ppGpp to these two RNAPs as non-specific or background binding, more than an order of magnitude weaker than the affinity of RNAP containing Site 1 or Site 2 for ppGpp.

We also tested ppGpp binding to another RNAP variant that previous work had shown responded very poorly to ppGpp *in vitro* using *in vitro* transcription as an assay (Ross et al., 2013). “RNAP M7” contains a four residue deletion at the N-terminus of ω, ω (Δ2–5), plus three other substitutions in β’ residues close to or within the Site 1 binding pocket. Surprisingly, this RNAP bound ppGpp with a K_d,app_ of 16.2 + 4.2 μM, only ~2-fold worse than the RNAPs with wild-type Site 1 or 2 (Supplementary Figure S4C). RNAPs containing a subset of the β’ substitutions present in M7 (e.g., β’K615A/R417A) were very defective in responding to ppGpp when assayed by *in vitro* transcription and by crosslinking with the zero-length crosslinker 6-thio ppGpp (Ross et al., 2013), yet displayed significant levels of binding in preliminary DRaCALA assays (Ross and Gourse, unpublished data). We suggest that crosslinking and function require very precise positioning of ppGpp in the binding pocket, but DRaCALA assays sometimes can detect binding modes that are non-functional. ppGpp binding to the M7 RNAP may represent such a non-functional binding mode.

### ppGpp Binds With Higher Affinity When RNAP Contains Both ppGpp Binding Sites

Even though Sites 1 and 2 are located ~60 Å apart on RNAP (Ross et al., 2016; Molodtsov et al., 2018) and have similar affinities for ppGpp, it was conceivable that having both sites would alter the overall ppGpp binding affinity. Therefore, we compared [^32^P]-ppGpp binding to RNAPs containing only Site 1 or Site 2 (Figure 2) with [^32^P]-ppGpp binding to an RNAP saturated with both ω and DksA (i.e., containing both sites; Figure 3A). For the RNAP with both ppGpp binding sites, we calculated the K_d,app_ from a plot in which the X-axis indicates the concentration of binding sites (twice the RNAP concentration). The data were fit for each of seven separate experiments, and the K_d,app_ values were averaged (Figure 3B). For the RNAP with both binding sites, the K_d,app_ was 3.3 ± 1.2 μM, a significant difference from the RNAPs with only one binding site (6.1 or 7.9 μM; *p* = 0.006 for both sites compared to Site 1 or *p* = 0.01 for both sites compared to Site 2). If the nominal concentration of RNAP were used on the X-axis as in Figure 2, rather than with the concentration of ppGpp binding sites, the K_d,app_ would be even tighter, 1.7 ± 1.2 μM. Thus, when plotted either way, there was a significant difference in the K_d,app_ of the enzyme for ppGpp with both sites vs. only one site.

The RNAP concentration in the DRaCALA reactions was 100–1,000-fold higher than the ppGpp concentration. Therefore, ppGpp could not fill both binding sites on an RNAP molecule at the same time. The measured binding affinity of the enzyme containing both sites must therefore represent an average of the affinities of ppGpp bound to one site or the other in the population of RNAPs. Nevertheless, we checked for cooperativity by comparing the fits using the equations for one-site and two-site saturation binding curves and the Hill equation. Both resulted in the same K_d,app_. The Hill coefficient was ~1, and the curve was not sigmoidal when the data were plotted on a linear scale (Supplementary Figure S5), consistent with a lack of cooperativity.

### DksA Binding to RNAP Enhances Binding of ppGpp to Site 1

Although there was no evidence for cooperativity, there was an increase in the affinity of ppGpp for wild-type RNAP containing DksA (both sites) compared to the affinity for RNAP with only one site (3.3 ± 1.2 μM for the RNAP containing both sites, compared to 6.1 ± 1.3 μM or 7.9 ± 1.3 μM for the RNAPs with either Site 1 or Site 2, respectively; Figures 2, 3). Since the DksA concentration was saturating in the DRaCALA reactions measuring binding of ppGpp to RNAP with both sites present, DksA was a candidate to explain the increase in affinity. That is, we hypothesized that DksA binding in the RNAP secondary channel might allosterically alter the binding environment of Site 1. To address this hypothesis, we utilized “separation of function” DksA variants, i.e., variants defective for ppGpp binding but competent for RNAP binding. DksA residues K98 and R129 both contact ppGpp directly (Ross et al., 2016; Molodtsov et al., 2018). DksA variants containing alanine substitutions at either of these positions are still able to bind to RNAP and reduce the lifetime of RNAP-promoter complexes (Ross et al., 2016), but they do not support ppGpp binding to Site 2, and they eliminate the effects of ppGpp on transcription by RNAPΔω (Ross et al., 2016). No binding was detected in DRaCALA experiments with RNAPΔω and either DksA-R129A or DksA-K98A, consistent with predictions for a complex lacking both binding sites for ppGpp (Supplementary Figures S6A,B).

**Figure 4:**
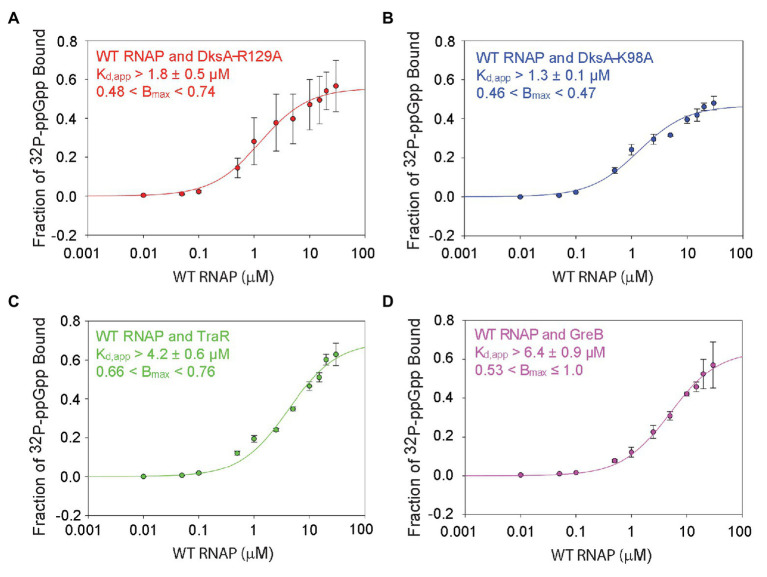
DksA enhances ppGpp binding to Site 1. DRaCALA measurements were performed with wild-type RNAP and the indicated secondary channel binding factors. **(A)** Wild-type RNAP with DksA-R129A. One-site saturation binding curves with data averaged from four independent experiments. K_d,app_ and B_max_ values, and error shown are averages from the values determined for each independent experiment. **(B)** Wild-type RNAP with DksA-K98A. Data from two independent experiments, as described in **(A)**. **(C)** Wild-type RNAP with TraR. Data from three independent experiments, as described in **(A)**. **(D)** Wild-type RNAP with GreB. Data from three independent experiments, as described in **(A)**.

Guanosine tetraphosphate bound to wild-type RNAP in the absence of DksA (i.e., to Site 1) with a K_d,app_ of 6.1 ± 1.3 μM (Figure 2). ppGpp bound to Site 1 in wild-type RNAP in the presence of DksA-R129A, with a K_d,app_ of ~1.8 ± 0.5 μM (Figure 4A), ~3-fold more tightly than to Site 1 without DksA (a statistically significant difference with a value of *p* = 0.001). In the presence of DksA-K98A, ppGpp bound to Site 1 with a K_d,app_ of ~1.3 ± 0.1 μM (Figure 4B), although the significance of this measurement is less certain because of the smaller number of replicates performed. Nevertheless, together these results indicate that ppGpp binds more tightly to Site 1 when DksA is bound to RNAP.

We also tested whether other secondary channel binding proteins increased the affinity of Site 1 for ppGpp. TraR is a distant homolog of DksA that is encoded by the F element (Blankschien et al., 2009). Although TraR is only half the size of DksA, it has an effect on transcription by itself that is as strong as the effect of DksA and ppGpp together (Gopalkrishnan et al., 2017). However, TraR lacks the residues in DksA that interact with ppGpp, and therefore ppGpp does not bind to RNAPΔω or increase the effect of TraR on transcription (Gopalkrishnan et al., 2017). The K_d,app_ for ppGpp binding to Site 1 in the wild-type RNAP-TraR complex was at least 4.2 ± 0.6 μM (Figure 4C; the curve did not plateau, so the affinity of ppGpp for Site 1 must be considered a lower estimate) vs. 6.1 ± 1.3 μM in the absence of TraR (Figure 2B). These K_d,app_ values for binding of ppGpp to Site 1 were not statistically different from each other. The TraR result is discussed further in the next section.

GreB is another secondary channel binding factor that does not function in conjunction with ppGpp (Rutherford et al., 2007; Lee et al., 2012). The K_d,app_ of ppGpp for the wild-type RNAP-GreB complex (i.e., containing Site 1) was ~6.4 ± 0.9 μM (Figure 4D), very similar to that in the absence of GreB (Figure 2), and ppGpp did not bind to the RNAPΔω-GreB complex (Supplementary Figure S6C). In summary, the mutant DksA proteins increased the affinity of ppGpp for Site 1, and these effects were specific, since other secondary channel binding factors did not increase the affinity of ppGpp for Site 1.

### Structural Basis for Effects of DksA on ppGpp Binding to Site 1

**Figure 5:**
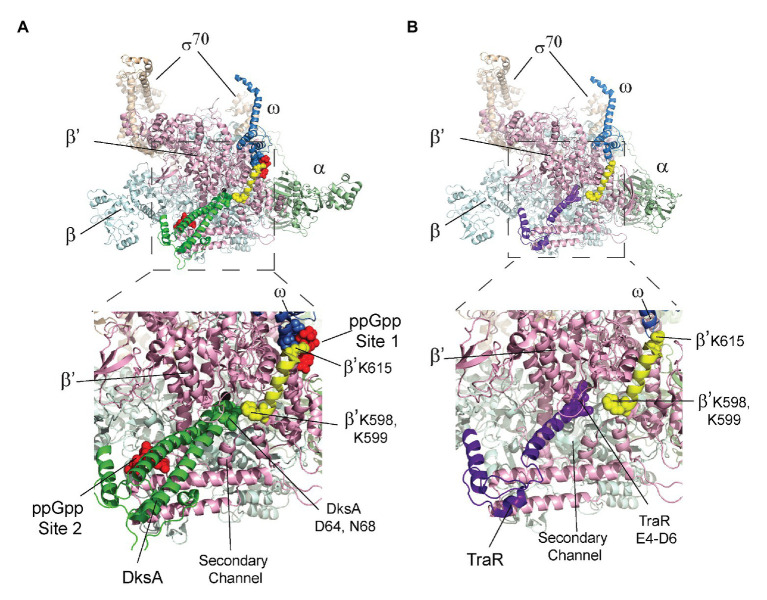
The β’ K598-K615 α-helix connects DksA and Site 1. The top image in each panel shows the crystal structure of RNAP with either **(A)** DksA or **(B)** TraR bound in the secondary channel. An expanded view of the boxed region in each panel is shown below the structures of RNAP. **(A)** X-ray crystal structures of wild-type RNAP holoenzyme with DksA and ppGpp soaked into the crystal (PDB 5VSW; Molodtsov et al., 2018). Coloring: α, green; β, cyan; β’, light pink; ω, gray; σ, tan; and ppGpp, red spheres. The β’ K598-K615 α-helix is yellow with K598, K599, and K615 shown as spheres. DksA is green with D64 and N68 shown as spheres. ω residues A2-T5 are shown as gray spheres. **(B)** RNAP holoenzyme with TraR (PDB 5W1S; Molodtsov et al., 2018). The β’ K598-K615 α-helix is yellow, and TraR is dark purple with residues E4, D6, and E7 near the TraR N-terminus shown as spheres.

The crystal structures of the RNAP-DksA-ppGpp complex and the RNAP-TraR complex (Molodtsov et al., 2018) provide a potential explanation for the observed effect of DksA, and not TraR, on ppGpp binding to Site 1. In the structures, DksA residues near its coiled coil tip (D64 and N68) are located 6–10 Å from β’ residues K598/K599. β’ K598 and K599 are at the N-terminus of a α-helix that extends to ppGpp binding Site 1 (Figure 5A). β’ residue K615, near the C-terminal end of this α-helix, interacts not only with ppGpp but also with residues in ω that bind to ppGpp. The proximity of DksA to β’-K598/K599 leads us to speculate that a DksA interaction with these residues in β’ might allosterically affect residues in Site 1, explaining the effect of DksA on ppGpp binding to Site 1 (Figure 4). Consistent with a direct interaction between DksA and K598/K599, an RNAP variant that contains alanine substitutions for K598 and K599 was partially resistant to DksA’s ability to shorten the lifetime of RNAP-promoter complexes (Vrentas, 2008).

In contrast, in the structure of the RNAP-TraR complex (Figure 5B; Molodtsov et al., 2018 Chen et al., 2019), there is a much greater separation (16–20 Å) between the N-terminus of TraR (corresponding to the coiled-coil tip region of DksA) and β’-K598/K599. We suggest that the increased separation between TraR and K598/K599 might explain the absence of a significant effect of TraR on ppGpp binding to Site 1 (Figure 4C). Thus, the structural information supports the biochemical data suggesting there is an allosteric effect of DksA on binding of ppGpp to Site 1.

### ω Levels Are Relatively Constant

DksA concentrations are constant throughout log phase and decrease only slightly in stationary phase (Rutherford et al., 2007). Therefore, changes in Site 2 binding of ppGpp are more likely a function of changes in ppGpp than DksA concentration, at least during exponential growth. In contrast, there is little information about the concentration of ω at different stages in growth or under different nutritional conditions. We reported previously that the magnitude of the effect of ppGpp on transcription *in vitro* increased slightly when RNAP was purified from cells in which ω was overproduced, suggesting that a small fraction of RNAP lacks ω in cells not overproducing ω (Vrentas et al., 2005).

To determine directly whether ω protein levels vary *in vivo*, we raised an antibody against ω and examined ω protein levels using Western blots. The antibody reacted with a band of the expected size in a strain with wild-type *rpoZ* but not one lacking *rpoZ* (Supplementary Figure S2B, compare lanes 1 and 6). Fortuitously, the anti-ω antibody also reacted with σ^70^. This cross-reactivity was verified using antibody to σ^70^ and ω, and Western blots with purified ω and σ^70^ (Supplementary Figures S2C,D).

**Figure 6:**
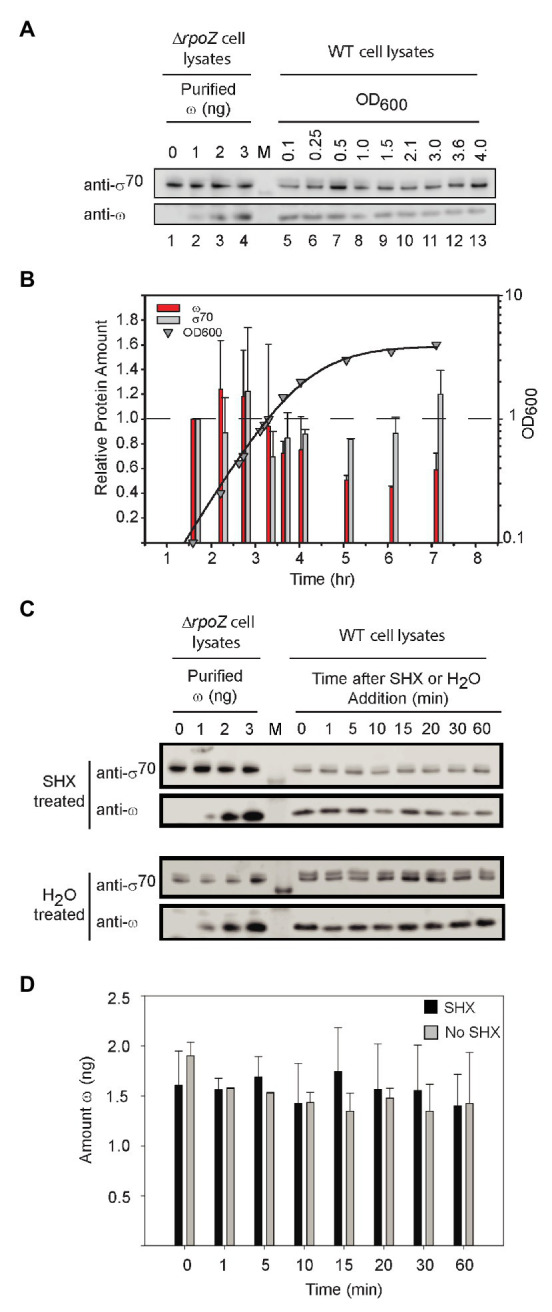
ω levels are relatively constant. **(A)** Representative Western blots using an anti-ω antibody. Purified ω was added to cell lysates from *ΔrpoZ* cells (RLG14044) to create a standard curve for estimation of the amount of ω (lanes 1–4). Wild-type cells were grown in LB, and lysates were collected at different OD_600_ for analysis of ω concentrations using an anti-ω antibody (lanes 5–13). Lane labeled M indicates molecular weight marker. The anti-ω antibody cross-reacted with σ^70^ (see Supplementary Figure S2). **(B)** The amounts of ω and σ^70^ at different times in growth were determined from experiments like that in **(A)** and are shown relative to the amounts present at an OD_600_ of 0.1 (first time point, ~1.5 h). Bars indicate the averages of three independent experiments, with error bars representing one SD from the mean. ω bars, red; σ^70^ bars, gray. ω decreased a maximum of ~2-fold, whereas σ^70^ (gray bars) remained relatively constant. **(C)** Representative Western blots with anti-ω antibody and cell lysates following treatment with serine hydroxamate (SHX) which starves cells for serine. Lanes labeled 0–3 indicate amounts of ω added FIGURE 6to cell lysates, lane labeled M indicates molecular weight marker, and lanes at right labeled 0–60 indicate minutes after SHX addition. **(D)** Average levels of ω in cells treated with SHX (black bars), compared to untreated cells (gray bars). ω bands in each Western blot were quantified using the ω standard curve derived from the same blot. The bars represent the average ω (in ng) for three separate experiments; error bars represent one SD from the mean.

As with DksA, the ω concentration remained relatively constant in cells growing exponentially in rich medium (LB), but it declined ~2-fold when cells transitioned to stationary phase (~OD_600_ = 1; Figures 6A,B). In contrast, the σ^70^ concentration was relatively constant. It is possible that the small decrease in ω levels during stationary phase could decrease the saturation of RNAP with ω and create a subpopulation of RNAP molecules defective in ppGpp binding to Site 1, but the changes in ω concentration are small and unlikely to be a major determinant of regulation by ppGpp during non-starvation conditions.

We also examined ω levels when ppGpp was induced to high concentration. After starvation of cells for serine aminoacyl tRNA by addition of serine hydroxamate (SHX; Figure 6C), ω concentrations were stable for at least 60 min, neither increasing nor decreasing well beyond the time needed for a typical stringent response (Figure 6D). We conclude that Site 1 occupancy by ppGpp is determined by changes in ppGpp concentration, not by changes in ω concentration, in both starved and unstarved cultures.

## Discussion

Although the regulatory role of ppGpp during the stringent response has been recognized for more than half a century (Cashel and Gallant, 1969), the mechanistic explanations for the effects of ppGpp on transcription are only now becoming clear. The recent discovery that there are two binding sites for ppGpp on RNAP has shed new light on the mechanisms of transcription regulation by ppGpp (Ross et al., 2016; Molodtsov et al., 2018). Here, we show that the two binding sites on RNAP have very similar intrinsic affinities for ppGpp, contradicting a model in which different affinities of the two sites for ppGpp increase the dynamic range of its effects on transcription during the stringent response. Instead, our data are more consistent with a model in which both binding sites reversibly bind ppGpp, are saturated to approximately the same extent when ppGpp concentrations are low, and become fully occupied at approximately the same time when ppGpp is induced to high levels.

We did not test binding of pppGpp (the pentaphosphate) to the two sites in RNAP. Available data indicate that, in *E. coli*, pppGpp is less abundant than ppGpp *in vivo* and that its effects on transcription *in vivo* and *in vitro*, particularly at Site 2, are less potent than those of ppGpp (Potrykus and Cashel, 2008; Mechold et al., 2013). Structures of the RNAP/DksA/ppGpp complex indicate that ppGpp at each site is partially solvent exposed (Zuo et al., 2013; Molodtsov et al., 2018), suggesting that each site could accommodate the additional phosphate group in pppGpp. However, it is not known whether the additional phosphate group could alter binding affinity.

Guanosine tetraphosphate binding to the two sites on RNAP can affect at least two different steps in transcription, impacting the kinetics of initiation by multiple mechanisms and resulting in different effects on transcriptional output from different promoters (Ross et al., 2016; Sanchez-Vazquez et al., 2019). For example, ppGpp binding to Site 2 can activate transcription from certain promoters whereas binding of ppGpp to Site 1 cannot (Ross et al., 2016; Gourse et al., 2018). In addition, it was also shown long ago that ppGpp affects transcription elongation *in vitro* (Kingston et al., 1981). Since those studies were performed without DksA, the effects on elongation were likely a result of binding to Site 1. Further studies will be needed to understand the mechanism(s) responsible for the effects of ppGpp on elongation, and which other factors play roles in these effects (see for example, Singh et al., 2016).

Given the concentration of ppGpp reported for non-starvation conditions in rich medium (~1–10 μM; Ryals et al., 1982), a concentration of RNAP *in vivo* of ~10 μM (Bremer and Dennis, 2008; Li et al., 2014), and the apparent binding affinities for ppGpp reported here, in a significant fraction of RNAP molecules it is likely that one or the other site would not be saturated with ppGpp. In the lower range of ppGpp concentrations (i.e., non-starvation conditions), when one site or the other is bound by ppGpp, this could result in some stochastic variation in regulation of transcription by ppGpp in subpopulations of RNAP molecules, creating some “bet-hedging” (Rocha et al., 2002). Stochasticity would be less impactful under starvation conditions when high ppGpp concentrations are present, and both ppGpp binding sites on all RNAP molecules would be saturated.

Depending on which step during transcription is affected, ppGpp function requires the presence of ω or DksA. However, since the concentrations of ω and DksA change little during exponential growth in *E. coli*, and no more than 2-fold in stationary phase, differences in the occupancies of the two ppGpp binding sites over time (and thus the impact of ppGpp on transcription) must result primarily from changes in ppGpp, not ω or DksA concentrations.

Interestingly, our data indicate that DksA increases the affinity of the wild-type RNAP for ppGpp (K_d,app_ = 3.3 ± 1.2 μM average affinity for the two binding sites on wild-type RNAP versus 6.1 ± 1.3 μM or 7.9 ± 1.3 μM, respectively, for RNAPs containing only Site 1 or 2). In addition, the affinity of ppGpp for the RNAPs in complex with the “separation of function” DksA variants is 1.3 ± 0.1–1.8 ± 0.5 μM. These results suggest that the increased affinity of Site 1 for ppGpp in the presence of DksA accounts for the reduced average affinity of wild-type RNAP for ppGpp relative to the affinity for either Site 1 or Site 2 alone. Thus, DksA allosterically enhances binding of ppGpp to Site 1, even though DksA and Site 1 are separated by ~30 Å at their position of closest approach (Figure 5). Although a limited number of secondary channel binding proteins was tested, we suggest this enhancement of ppGpp affinity for Site 1 is unique to DksA (Figure 4). The physiological consequence, if any, of the enhancement in affinity of Site 1 for ppGpp by DksA remains to be determined.

Finally, the concentrations of ppGpp observed during the stringent response appear to be much higher than needed for full occupancy of the two sites on RNAP. Although the high concentrations undoubtedly evolved in part to increase the kinetics of ppGpp occupancy of RNAP, some 70 proteins in *E. coli* bind ppGpp, only a subset of which bind ppGpp with an affinity as high as RNAP (Pao and Dyess, 1981; Hou et al., 1999; Kanjee et al., 2011a,b; Zhang et al., 2018; Anderson et al., 2019; Wang et al., 2019). We suggest that the high levels of ppGpp produced during severe starvations could be needed for ppGpp to bind to the proteins with lower affinity ppGpp binding sites.

## Data Availability Statement

All datasets presented in this study are included in the article/Supplementary Material.

## Author Contributions

AM designed, performed, and analyzed experiments and wrote the paper. WR analyzed experiments and wrote the paper. DT performed and analyzed experiments. RG designed and analyzed experiments and wrote the paper. All authors contributed to the article and approved the submitted version.

### Conflict of Interest

The authors declare that the research was conducted in the absence of any commercial or financial relationships that could be construed as a potential conflict of interest.

## References

[ref1] Anderson B. W. Liu K. Wolak C. Dubiel K. She F. Satyshur K. A.. (2019). Evolution of (p)ppGpp-HPRT regulation through diversification of an allosteric oligomeric interaction. Elife 8:e47534. 10.7554/eLife.47534, PMID: 31552824 PMC6783271

[ref2] Blankschien M. D. Potrykus K. Grace E. Choudhary A. Vinella D. Cashel M.. (2009). TraR, a homolog of a RNAP secondary channel interactor, modulates transcription. PLoS Genet. 5:e1000345. 10.1371/journal.pgen.1000345, PMID: 19148274 PMC2613031

[ref3] Bremer H. Dennis P. P. (2008). Modulation of chemical composition and other parameters of the cell at different exponential growth rates. EcoSal Plus 3. 10.1128/ecosal.5.2.3, PMID: 26443740

[ref4] Cashel M. Gallant J. (1969). Two compounds implicated in the function of the RC genes of *Escherichia coli*. Nature 221, 838–841. 10.1038/221838a0, PMID: 4885263

[ref6] Chen J. Gopalkrishnan S. Chiu C. Chen A. Y. Campbell E. A. Gourse R. L.. (2019). *E. coli* TraR allosterically regulates transcription initiation by altering RNA polymerase conformation. Elife 8:e49375. 10.7554/eLife.49375, PMID: 31841111 PMC6970531

[ref7] Durfee T. Hansen A. M. Zhi H. Blattner F. R. Jin D. J. (2008). Transcription profiling of the stringent response in *Escherichia coli*. J. Bacteriol. 190, 1084–1096. 10.1128/JB.01092-07, PMID: 18039766 PMC2223561

[ref9] Gentry D. R. Burgess R. R. (1989). *rpoZ*, encoding the omega subunit of *Escherichia coli* RNA polymerase, is in the same operon as spoT. J. Bacteriol. 171, 1271–1277. 10.1128/jb.171.3.1271-1277.1989, PMID: 2646273 PMC209740

[ref8] Gentry D. Xiao H. Burgess R. R. Cashel M. (1991). The omega subunit of *Escherichia coli* K-12 RNA polymerase is not required for stringent RNA control in vivo. J. Bacteriol. 173, 3901–3903. 10.1128/jb.173.12.3901-3903.1991, PMID: 1711031 PMC208023

[ref10] Gopalkrishnan S. Ross W. Chen A. Y. Gourse R. L. (2017). TraR directly regulates transcription initiations by mimicking the combined effects of the global regulators DksA and ppGpp. Proc. Natl. Acad. Sci. U. S. A. 114, E5539–E5548. 10.1073/pnas.1704105114, PMID: 28652326 PMC5514744

[ref11] Gourse R. L. Chen A. Y. Gopalkrishnan S. Sanchez-Vazquez P. Myers A. Ross W. (2018). Transcriptional responses to ppGpp and DksA. Annu. Rev. Microbiol. 72, 163–184. 10.1146/annurev-micro-090817-062444, PMID: 30200857 PMC6586590

[ref12] Hauryliuk V. Atkinson G. C. Murakami K. S. Tenson T. Gerdes K. (2015). Recent functional insights into the role of (p)ppGpp in bacterial physiology. Nat. Rev. Microbiol. 13, 298–309. 10.1038/nrmicro3448, PMID: 25853779 PMC4659695

[ref13] Hill A. V. (1910). The possible effects of the aggregation of the molecules of haemoglobin on its dissociation curves. J. Physiol. 40, iv–vii.

[ref14] Hou Z. Cashel M. Fromm H. J. Honzatko R. B. (1999). Effectors of the stringent response target the active site of *Escherichia coli* adenylosuccinate synthetase. J. Biol. Chem. 274, 17505–17510. 10.1074/jbc.274.25.17505, PMID: 10364182

[ref15] Irving S. E. Corrigan R. M. (2018). Triggering the stringent response: signals responsible for activating (p)ppGpp synthesis in bacteria. Microbiology 164, 268–276. 10.1099/mic.0.000621, PMID: 29493495

[ref16] Kanjee U. Gutsche I. Alexopoulos E. Zhao B. Y. El Bakkouri M. Thibault G.. (2011a). Linkage between the bacterial acid stress and stringent responses: the structure of the inducible lysine decarboxylase. EMBO J. 30, 931–944. 10.1038/emboj.2011.5, PMID: 21278708 PMC3049219

[ref17] Kanjee U. Gutsche I. Ramachandran S. Houry W. A. (2011b). The enzymatic activities of the *Escherichia coli* basic aliphatic amino acid decarboxylases exhibit a pH zone of inhibition. Biochemistry 50, 9388–9398. 10.1021/bi201161k, PMID: 21957966

[ref18] Kingston R. E. Nierman W. C. Chamberlin M. J. (1981). A direct effect of guanosine tetraphosphate on pausing *Escherichia coli* RNA polymerase during RNA chain elongation. J. Biol. Chem. 256, 2787–2797. PMID: 7009598

[ref19] Krasny L. Gourse R. L. (2004). An alternative strategy for bacterial ribosome synthesis: *Bacillus subtilis* rRNA transcription regulation. EMBO J. 23, 4473–4483. 10.1038/sj.emboj.7600423, PMID: 15496987 PMC526457

[ref20] Lee J. -H. Lennon C. W. Ross W. Gourse R. L. (2012). Role of the coiled-coil tip of *Escherichia coli* DksA in promoter control. J. Mol. Biol. 416, 503–517. 10.1016/j.jmb.2011.12.028, PMID: 22200485 PMC3288215

[ref21] Li G. -W. Burkhardt D. Gross C. Weissman J. S. (2014). Quantifying absolute protein synthesis rates reveals principles underlying allocation of cellular resources. Cell 157, 624–635. 10.1016/j.cell.2014.02.033, PMID: 24766808 PMC4006352

[ref22] Liu K. Bittner A. N. Wang J. D. (2015). Diversity in (p)ppGpp metabolism and effectors. Curr. Opin. Microbiol. 24, 72–79. 10.1016/jmib.2015.01.012, PMID: 25636134 PMC4380541

[ref23] Mechold U. Potrykus K. Murphy H. Murakami K. S. Cashel M. (2013). Differential regulation by ppGpp versus pppGpp in *Escherichia coli*. Nucleic Acids Res. 41, 6175–6189. 10.1093/nar/gkt302, PMID: 23620295 PMC3695517

[ref24] Molodtsov V. Sineva E. Zhang L. Huang X. Cashel M. Ades S. E.. (2018). Allosteric effector ppGpp potentiates the inhibition of transcript initiation by DksA. Mol. Cell 69, 828.e5–839.e5. 10.1016/j.molcel.2018.01.035, PMID: 29478808 PMC5837818

[ref25] Pao C. C. Dyess B. T. (1981). Effect of unusual guanosine nucleotides on the activities of some *Escherichia coli* cellular enzymes. Biochim. Biophys. Acta 677, 358–362. 10.1016/0304-4165(81)90247-6, PMID: 6117328

[ref26] Paul B. J. Barker M. M. Ross W. Schneider D. A. Webb C. Foster J. W.. (2004). DksA: a critical component of the transcription initiation machinery that potentiates the regulation of rRNA promoters by ppGpp and the initiating NTP. Cell 118, 311–322. 10.1016/j.cell.2004.07.009, PMID: 15294157

[ref27] Paul B. J. Berkmen M. B. Gourse R. L. (2005). DksA potentiates direct activation of amino acid promoters by ppGpp. Proc. Natl. Acad. Sci. U. S. A. 102, 7823–7828. 10.1073/pnas.0501170102, PMID: 15899978 PMC1142371

[ref28] Potrykus K. Cashel M. (2008). (p)ppGpp: still magical? Annu. Rev. Microbiol. 62, 35–51. 10.1146/annurev.micro.62.081307.162903, PMID: 18454629

[ref29] Rocha E. P. C. Matic I. Taddei F. (2002). Over-representation of repeats in stress response genes: a strategy to increase versatility under stress conditions? Nucleic Acids Res. 30, 1886–1894. 10.1093/nar/30.9.1886, PMID: 11972324 PMC113848

[ref30] Roelofs K. G. Wang J. Sintim H. O. Lee V. T. (2011). Differential radial capillary action of ligand assay for high-through put detection of protein-metabolite interactions. Proc. Natl. Acad. Sci. U. S. A. 108, 15528–15533. 10.1073/pnas.1018949108, PMID: 21876132 PMC3174574

[ref32] Ross W. Sanchez-Vazquez P. Chen A. Y. Lee J. -H. Burgos H. L. Gourse R. L. (2016). ppGpp binding to a site at the RNAP-DksA interface accounts for its dramatic effects on transcription initiation during the stringent response. Mol. Cell 62, 811–823. 10.1016/j.molcel.2016.04.029, PMID: 27237053 PMC4912440

[ref31] Ross W. Vrentas C. E. Sanchez-Vazquez P. Gaal T. Gourse R. L. (2013). The magic spot: a ppGpp binding site on *E. coli* RNA polymerase responsible for regulation of transcription initiation. Mol. Cell 50, 420–429. 10.1016/j.molcel.2013.03.021, PMID: 23623682 PMC3654024

[ref33] Rutherford S. T. Lemke J. J. Vrentas C. E. Gaal T. Ross W. Gourse R. L. (2007). Effects of DksA, GreA, and GreB on transcription initiation: insights into the mechanisms of factors that bind in the secondary channel of RNA polymerase. J. Mol. Biol. 366, 1243–1257. 10.1016/j.jmb.2006.12.013, PMID: 17207814 PMC1839928

[ref34] Ryals J. Little R. Bremer H. (1982). Control of RNA synthesis in *Escherichia coli* after a shift to higher temperature. J. Bacteriol. 151, 1425–1432. 10.1128/JB.151.3.1425-1432.1982, PMID: 6179925 PMC220424

[ref35] Sanchez-Vazquez P. Dewey C. N. Kitten N. Ross W. Gourse R. L. (2019). Genome-wide effects on *Escherichia coli* transcription from ppGpp binding to its two sites on RNA polymerase. Proc. Natl. Acad. Sci. U. S. A. 116, 8310–8319. 10.1073/pnas.1819682116, PMID: 30971496 PMC6486775

[ref36] Singh N. Bubunenko M. Smith C. Abbott D. M. Stringer A. M. Shi R.. (2016). SuhB associates with nus factors to facilitate 30S ribosome biogenesis in *Escherichia coli*. mBio 7:e00114. 10.1128/mBio.00114-16, PMID: 26980831 PMC4807359

[ref37] Traxler M. F. Summer S. M. Nguyen H. T. Zacharia V. M. Hightower G. A. Smith J. T.. (2008). The global ppGpp-mediated stringent response to amino acid starvation in *Escherichia coli*. Mol. Microbiol. 68, 1128–1148. 10.1111/j.1365-2958.2008.06229.x, PMID: 18430135 PMC3719176

[ref45] Varik V. Oliveira S. R. A. Hauryliuk V. Tenson T. (2017). HPLC-based quantification of bactgerial housekeeping nucleotides and alarmone messengers ppGpp and pppGpp. Sci. Rep. 7:11022. 10.1038/s41598-017-10988-6, PMID: 28887466 PMC5591245

[ref38] Vrentas C. E. (2008). A study of the requirements for the response of *Escherichia coli* RNA polymerase to guanosine tetraphosphate (ppGpp). University of Wisconsin-Madison.

[ref40] Vrentas C. E. Gaal T. Ross W. Ebright R. H. Gourse R. L. (2005). Response of RNA polymerase to ppGpp: requirement for the omega subunit and relief of this requirement by DksA. Genes Dev. 19, 2378–2387. 10.1101/gad.1340305, PMID: 16204187 PMC1240046

[ref41] Wang B. Dai P. Ding D. Del Rosario A. Grant R. A. Pentelute B. L.. (2019). Affinity-based capture and identification of protein effectors of the growth regulator ppGpp. Nat. Chem. Biol. 15, 141–150. 10.1038/s41589-018-0183-4, PMID: 30559427 PMC6366861

[ref42] Xiao H. Kalman M. Ikehara K. Zemel S. Glaser G. Cashel M. (1991). Residual guanosine 3',5'-bispyrophosphate synthetic activity of relA null mutants can be eliminated by spoT null mutations. J. Biol. Chem. 266, 5980–5990. PMID: 2005134

[ref43] Zhang Y. Zbornikova E. Rejman D. Gerdes K. (2018). Novel (p)ppGpp binding and metabolizing proteins in *Escherichia coli*. mBio 9, e02188–e02217. 10.1128/mBio.02188-17, PMID: 29511080 PMC5845004

[ref44] Zuo Y. Wang Y. Steitz T. A. (2013). The mechanism of *E. coli* RNA polymerase regulation by ppGpp is suggested by the structure of their complex. Mol. Cell 50, 430–436. 10.1016/j.molcel.2013.03.020, PMID: 23623685 PMC3677725

