# Supplementary material for: Guanosine Tetraphosphate Has a Similar Affinity for Each of Its Two Binding Sites on Escherichia coli RNA Polymerase
Source: Front Microbiol. 2020 Nov 5;11:587098. doi: 10.3389/fmicb.2020.587098 (PMC7676912; doi:10.3389/fmicb.2020.587098)
Supplement: Supplementary file 1 [file Data_Sheet_1.pdf]

SUPPLEMENTAL DATA

Expanded Materials and Methods

Protein Purification.

RNAP. Wild-type RNAP saturated with ω was purified from BL21 DE3 cells containing the multi-subunit RNAP overexpression vector pRLG9960 (see Table S1) and the ω overexpression plasmid pCDFω. Purification included PEI precipitation, ammonium sulfate precipitation, Ni-NTA agarose chromatography, and heparin sepharose chromatography as described previously (Vrentas et al., 2008; Ross et al., 2013). For elution from the Ni-NTA column, an imidazole step gradient was used, with collection of 1 ml fractions starting at 300 mM imidazole. For elution from the heparin column, an NaCl step gradient was used with collection of 1 ml fractions starting at 600 mM NaCl. RNAPΔω was purified from BL21 DE3 *ΔrpoZ::kan* cells containing pRLG9960 using the same protocol listed above. Wild-type RNAP and RNAPΔω were dialyzed into DRaCALA Buffer (15 mM Tris, pH 8.0, 170 mM NaCl, 10 mM $MgCl_2$, 1 mM DTT, 1% glycerol) overnight, flash frozen on dry ice and ethanol, and stored at -80°C. Representative gels of wild-type RNAP and RNAPΔω are shown in Figure S1A and S1B. Pooled fractions are indicated, and the final protein concentration was determined by Bradford assay. For the wild-type RNAP and RNAPΔω purification, the RNAP subunits are labeled. A band in the RNAPΔω purifications using RLG14698 that migrated similar to ω did not react with the anti-ω antibody in Western blots (Figures S2B and C). All preparations of RNAP from strains lacking ω, RNAPΔω (regardless of strain background) displayed more proteolysis than preparations of wild-type RNAP. The band of similar size to ω may be a proteolytic product of an unrelated protein that migrated like ω. In any case, we conclude that RNAPΔω preparations lack ω.

DksA. Wild-type DksA and DksA-R129A were purified by a modification of the method described previously (Paul et al., 2004) using Ni-NTA agarose chromatography, an imidazole step gradient, and elution with 300 mM imidazole. The N-terminal His-tag was removed by cleavage with biotinylated thrombin (Novagen). Thrombin and the cleaved His-tag were separated from DksA with Ni-NTA agarose and Streptavidin agarose. The DksA proteins were then dialyzed into DRaCALA buffer overnight, flash frozen on dry ice and ethanol, and stored at -80°C. DksA-K98A was purified using the above method, but after dialysis into 10 mM Tris-Cl, pH 8.0, 100 mM NaCl, 1 mM DTT, and 50% glycerol, the protein was stored at -80°C. Prior to DRaCALA experiments, DksA-K98A was dialyzed into DRaCALA buffer, flash frozen with dry ice and ethanol, and stored at -80°C.

GreB. GreB was purified from BL21 DE3 cells containing the GreB overexpression plasmid pMO1.4His (Koulich *et al.*, 1997) using Ni-NTA agarose chromatography (Qiagen) as described (Lee et al., 2012). The N-terminal His-tag was removed as with DksA above, and GreB was then dialyzed into 10 mM Tris-HCl, pH 8.0, 100 mM NaCl, 1 mM DTT, and 50% glycerol and stored at -80°C.

TraR. TraR was purified from BL21 DE3 *ΔdksA::Tn10* cells containing the His10-SUMO-TraR overexpression plasmid pRLG15142 (Chen et al., 2020) using Ni-NTA agarose

chromatography (Qiagen). The N-terminal SUMO-tag was removed by cleavage with the protease Ulp1 (1:400 Ulp1:TraR, w/w) (Invitrogen). The His10-SUMO tag was separated by Ni-NTA agarose. TraR was collected in the flow-through and then dialyzed into a buffer containing 10 mM Tris-HCl pH 8.0, 250 mM NaCl, 0.1 mM EDTA, 2 mM DTT, and 50% glycerol. For DRaCALA experiments, TraR was removed from storage at -80°C, buffer-exchanged into the DRaCALA buffer by two successive exchanges of 2 ml each (for a total of 10-fold the original vol of TraR in storage buffer) using a 3500 MWCO Amicon Ultra-4 centrifugal filter unit (UFC800324; Millipore) at 4°C, and used immediately for DRaCALA assays.

ω. To purify ω for antibody development, BL21 DE3 cells containing pET23a-His10-SUMO-*rpoZ* (RLG15371) were diluted into LB medium from an overnight culture to OD_{600} ~0.01 and grown to OD_{600} ~0.6 at 37 °C (mid-log). 1 mM isopropyl-β-D-thiogalactoside (IPTG) was then added, the culture was grown for another 3 hr, and the cells were harvested by centrifugation and resuspended in SUMO lysis buffer (20 mM Tris, pH 8.0, 0.5 M NaCl, 10 % glycerol, 0.5 mM BME, 0.1 mM PMSF). Lysozyme (0.2 mg/ml final concentration) and 100 ul HALT Protease Inhibitor Cocktail (Thermo Fisher) was then added on ice for 10 min, and cells were lysed by sonication on ice and centrifuged for 30 min at 15,000 RPM to clear the lysate. Imidazole was added to a final concentration of 10 mM, the lysate was loaded onto a Ni-NTA (Qiagen) gravity column, and washed with 10 ml of SUMO lysis buffer (see above) containing 10 mM imidazole. After a second wash with 3 ml SUMO lysis buffer containing 150 mM imidazole, the column was eluted with SUMO lysis buffer containing 300 mM imidazole. 10 1 ml fractions were analyzed by SDS-PAGE, and fractions containing His10-SUMO-ω were pooled.

Because σ^{70} was detected in the ω preparation (Figure S1E, lane 3, His10-SUMO-ω Elute – Nickel Column 1), ω was purified further. The pooled fractions were diluted to 100 mM imidazole and run on a second Ni-NTA gravity column, as described above. Peak fractions were mixed with His8-Ulp1 (SUMO protease) at a ratio of 1:400 (mg Ulp1: mg protein), dialyzed overnight in SUMO cleavage buffer (20 mM Tris, pH 8.0, 0.15 M NaCl, 5 % glycerol, 1 mM BME), and the dialyzed mixture was then run on a Ni-NTA (Qiagen) column equilibrated with the SUMO cleavage buffer. His10-SUMO and His8-Ulp1 remained bound to the column, and the flow through was collected. The cleavage of the ω fraction was visualized on a 12% polyacrylamide Nu-PAGE gel (Invitrogen) (Figure S2A), and the concentration of protein was determined by Bradford assay (Bio-Rad).

Measurement of ppGpp Binding to RNAP by DRaCALA

Binding of ppGpp to RNAP, RNAP-DksA, RNAP-TraR, or RNAP-GreB complexes was measured by the Differential Radial Capillary Action of Ligand Assay (DRaCALA; Roelofs et al., 2011), adapted for [^{32}P]-ppGpp (Ross et al., 2016). All binding reactions were performed on core RNAP (α2ββ'ω). The ppGpp binding sites are distant from the position of σ^{70}, and previous data suggested that σ did not affect ppGpp binding to RNAP (Ross et al. 2016 and data not shown). By omitting σ, we also avoided the potential that different RNAP preparations might contain different amounts of σ that might affect quantitative comparisons of binding.

[^{32}P]-ppGpp was synthesized and purified as described previously (Ross et al., 2016) using γ-[^{32}P]-ATP and GDP (Amresco) as the substrate and a crude ribosome fraction as the source of

RelA activity. [^{32}P]-ppGpp was purified by PEI cellulose thin layer chromatography in 1.5 M KH_2PO_4 pH 3.4, eluted from the TLC plate in 4 M LiCl, precipitated, and then stored in aliquots at -80°C as described (Ross et al., 2013).

All reactions contained 15 mM Tris-HCl pH 8.0, 170 mM KCl, 10 mM $MgCl_2$, 1 mM DTT, and 1% glycerol. For measuring ppGpp binding to Site 1 in the absence of Site 2, 15 µl reactions contained ~5 nM [^{32}P]-ppGpp and 0.01 to 30 µM wild-type RNAP (i.e. no DksA). To measure ppGpp binding to Site 2 in the absence of Site 1, the reactions (15 µl) contained ~5 nM [^{32}P]-ppGpp, 0.01 to 5 µM RNAPΔω, the wild-type DksA concentration was 25 µM. When the RNAPΔω concentration was 10 to 30 µM, the DksA concentration was 3-fold greater (30 to 90 µM) than the RNAPΔω concentration in order to ensure that DksA was in stoichiometric excess. For measuring ppGpp binding to RNAP with both sites, the reactions contained ~5 nM [^{32}P]-ppGpp, 0.01 to 30 µM RNAP, and 25 to 90 µM DksA, as described above.

Reactions were incubated at 23°C for 10 min, and triplicate 4.0 µl aliquots of each sample were spotted on dry Protran BA85 nitrocellulose membrane filters. The filters were then dried and phosphorimaged. The ^{32}P bound to the central spot was determined, corrected for unbound ligand (see Roelofs et al., 2011). Radioactivity in the outer ring of the spot was not included in determining background correction values. The amount of bound [^{32}P]-ppGpp was expressed as a percentage of total counts in the entire spot (Fraction [^{32}P]-ppGpp Bound; Roelofs et al., 2011). To determine the ppGpp binding affinity for Site 1, Site 2, and both sites, the fraction of [^{32}P]-ppGpp bound was determined independently for each of 7 biological replicates and then averaged and fit to a one-site binding curve. The average fraction of [^{32}P]-ppGpp bound from each independent experiment was calculated from WT RNAP-[^{32}P]-ppGpp complexes for Site 1, from RNAPΔω-WT DksA-[^{32}P]-ppGpp complexes for Site 2, and from WT RNAP-WT DksA-[^{32}P]-ppGpp complexes for both sites together, where: $y = (B_{max} * x)/(K_d + x)$.

The B_{max} was allowed to float to achieve the best-fit curve. The experimental $K_{d,app}$ values were averaged to determine an overall $K_{d,app}$, and the standard error for the mean was determined. For DRaCALA measurements of ppGpp binding to Site 2 alone, RNAP was purified from a strain lacking ω (*rpoZ,* Figure S2). For DRaCALA measurements of ppGpp binding to Site 1 alone, RNAP was purified from a strain lacking DksA (*ΔdksA*). For DRaCALA measurements of ppGpp binding to RNAP without either site, RNAP was purified from three different strains, one lacking ω (RLG14698), one containing only ω(Δ2-5) (RLG14751), and one containing ω(Δ2-5) and 3 substitutions in the β' subunit ("M7"; RLG14293), and DksA was not included in the binding reactions (Figure S4). The B_{max} was set to 1.0. Previous experiments have shown that eliminating both ω and DksA abolishes ppGpp-dependent inhibition of RNAP (Ross et al., 2016). Therefore, we consider the low level of binding observed by DRaCALA in the absence of both ω and DksA as the non-specific, background level of binding.

For measuring ppGpp binding to Site 1 in the presence DksA-K98A, DksA-R129A, TraR, or GreB, 15 µl reactions contained ~5 nM [^{32}P]-ppGpp, 0.01 – 30 µM WT RNAP, and 25 – 90 µM of secondary-channel binding protein, as described for DksA above. Four biological replicates were measured with DksA-R129A, 2 with DksA-K98A, 3 with TraR, and 3 with GreB at different RNAP concentrations, and the results were fit to a one-site binding curve using the equation above to determine the ppGpp binding affinity, treating fraction of [^{32}P]-ppGpp bound separately for each experiment, as described above. The B_{max} was allowed to float to achieve the best-fit curve. The separate $K_{d,app}$ values were then averaged to determine an overall $K_{d,app}$, and the standard error for the mean was determined. For curves that did not

reach saturation, (WT RNAP with DksA-R129A, DksA-K98A, TraR, or GreB), the best-fit curve was still calculated with the B_{max} allowed to float, but $K_{d,app}$ are provided using greater than or less than symbols. The error provided is the standard error of the mean.

To determine whether there was evidence for cooperativity in ppGpp binding, we created a linear plot of the data from Figure 3 (reactions containing the wild-type RNAP and DksA) rather than a semi-log plot (see Figure S5 and legend). We show fits of the data using a one site or a two site saturation equation, or a sigmoidal dose-response equation. The data fit the one site and two site equations equally well, but did not show cooperativity according to the Hill equation (the Hill coefficient was ~ 1). In addition, the curve was not sigmoidal when the data were plotted on a linear scale. Both the one-site saturation binding curve and the Hill equation gave the same $K_{d,app}$.

Western Blots.

Sera from ten rabbits were screened for cross-reactivity to *E. coli* proteins, and the rabbit with the lowest cross-reactivity to low molecular weight proteins was selected for antibody production. 250 μg of purified ω was injected intravenously, followed by four additional injections with 125 μg of ω at 21 day intervals. Sera were harvested 10 days after injections 2 to 5. The final bleed was performed at 91 days after the initial injection.

For verification of anti-ω reactivity from rabbit sera of the final bleed, wild-type cells (RLG14475) and *ΔrpoZ* cells (RLG 14044) were diluted into LB medium from an overnight culture to OD_{600} ~0.01, grown to OD_{600} ~0.6 at 37°C. (mid-log). The cells were then harvested by centrifugation, lysed using 2x SDS loading buffer (4% SDS, 20% glycerol, 200 mM DTT, 0.01% bromophenol blue, 0.1 M Tris-HCl, pH 8.0) in a volume normalized to OD_{600}, and stored at -20°C. Lysates were thawed, heated at 95°C for 15 min, 5 μl of each lysate was examined on a 4-12% Nu-PAGE gel (Invitrogen), and proteins were transferred to Protran BA-85 membranes (GE Life Sciences). Western blots were performed using standard protocols (Bio-Rad) with rabbit polyclonal anti-ω serum (1:10,000 dilution), an anti-rabbit IgG HRP-conjugated secondary antibody (1:10,000 dilution, Product number: W401B; Promega) and SuperSignal West Dura Extended Duration Substrate (Thermo Fisher). HRP signal was visualized on x-ray film after 30 min of exposure (Figure S2C). Several proteins in the *E. coli* lysate cross-reacted with the anti-ω antibody, including σ^{70} (verified by Western blot analysis of the same lysate with anti-σ^{70} (Figures S2B, C, and D). However, the ω band was easily distinguishable from other bands in the lysate.

For quantitative Western blots from cells grown in LB, wild-type cells (RLG14475) were diluted into fresh LB from an overnight culture to OD_{600} ~0.01 and grown at 37 °C. At specific times, 0.5 OD_{600} of cells were pelleted, the supernatant was removed, and cells were resuspended in 100 μl 2x SDS loading buffer. The lysed cells were then stored at -20°C. For serine hydroxamate-treated cells, wild-type cells (RLG14475) were diluted from an overnight culture into fresh LB to OD_{600} ~0.01 and grown at 37°C to an OD_{600} of ~0.3. The culture was then divided into two flasks, one of which was treated with serine hydroxamate to a final concentration of 1 mg/ml. An equal volume of water was added to the other flask as a control. Samples were removed every 5-10 min for 60 min, and cells were harvested as described above. The lysed cells were stored at -20°C until thawed and heated to 95°C for 15 min. 5 μl of each cell lysate was examined on 4-12% Nu-PAGE gels (Invitrogen) and transferred to Protran BA-85 membranes (GE Life Sciences). For some Western blots (e.g. Figures 6 and S2B), known quantities of purified ω were added to the 0.025 ODs of cell lysates from the *ΔrpoZ*

strain (RLG14044), and the HRP signal was visualized using a Li-Cor Odyssey Fc and quantified using Li-Cor quantitation software.

Table S1. Strains and Plasmids used in this study.

**Strain**	**Genotype**	**Reference**
RLG7075	BL21 DE3, *dksA::Tn10*	Paul et al., 2004
RLG7804	BL21 DE3 (pNH2-His6-GreB)	Koulich et al., 1997
RLG7817	BL21 DE3 (pCDFω)	Vrentas et al., 2005
RLG12113	*rpoZ::kan* (Keio Strain JW3624)	Baba et al., 2006
RLG12115	BL21 DE3, *rpoZ::kan*	Ross et al., 2013
RLG13165	BL21 DE3 *dksA::tet* (pET33-His10-HMK-*dksA*)	Lee et al., 2012
RLG13173	BL21 DE3 *dksA::tet* (pET33-His10-HMK-*dksA-K98A*)	Ross et al., 2016
RLG13179	BL21 DE3 *dksA::tet* (pET33-His10-HMK-*dksA-R129A*)	Ross et al., 2016
RLG14044	MG1655 *rpoZ::kan*	This Work
RLG14235	DH5α (pET23a-His10-SUMO)	Henry et al., in press
RLG14293	BL21 DE3, *rpoZ::kan,* (pCDFωΔ2-5) (pIA299 *rpoC*-His6 R362A, R417A, K615A)	This Work
RLG14475	MG1655	Sanchez-Vazquez et al., 2019
RLG14698	BL21 DE3, *rpoZ::kan* (pRLG9960 = *rpoA*, *rpoB*, *rpoC*-His10 (RNAP overexpression vector, derivative of pIA299)	This Work
RLG14751	RLG14698 (pCDFωΔ2-5)	This Work
RLG14763	BL21 DE3 (pRLG9960, pCDFω)	This Work
RLG15142	BL21 DE3, *dksA::Tn10* (pET23a-SUMO-His10-*traR*, ΔMet1*)*	Chen et al., 2019
RLG15335	BL21 DE3 Δ*dksA* (pRLG9960 pCDFω)	This Work
RLG15371	BL21 DE3 (pET23a-His10-SUMO-*rpoZ* ΔMet1)	This Work

Table S2. Summary of ppGpp binding data

ppGpp Binding Site+	RNA Polymerase	Secondary Channel Factor	$K_{d,app}$ (µM)	Figure
Site 1 only	WT RNAP	--	6.1 ± 1.3	2B
	WT RNAP	DksA R129A	1.8 ± 0.5	4A
	WT RNAP	DksA K98A	1.3 ± 0.1	4B
	WT RNAP	TraR	> 4.2 ± 0.6	4C
	WT RNAP	GreB	> 6.4 ± 0.9	4D
Site 2 only	RNAPΔω	WT DksA	7.9 ± 1.3	2D
Both Sites 1 and 2	WT RNAP	WT DksA	3.3 ± 1.2*	3B
No Sites (mutations in Sites 1 and 2)	RNAPΔω	--	> 100	S4A
	RNAPΔω + ωΔ2-5	--	> 100	S4B
	RNAPΔω (β'R362A, β'R417A, β'R615A) + ωΔ2-5 ("M7")	--	16.2 ± 4.2	S4C
	RNAPΔω	DksA R129A	No Binding	S6A
	RNAPΔω	DksA K98A	No Binding	S6B
	RNAPΔω	GreB	No Binding	S6C

* A $K_{d,app}$ = 3.3 ± 1.2 µM reflects fitting data to one-sight saturation binding curve with the number of ppGpp binding sites plotted on the x-axis. If the concentration of WT RNAP is instead plotted on the x-axis, the $K_{d,app}$ = 1.7 ± 1.2 µM. (See Results)
+ ppGpp sites are as reported in Ross et al (2013); Ross et al. (2016); Sanchez-Vazquez et al, (2019).

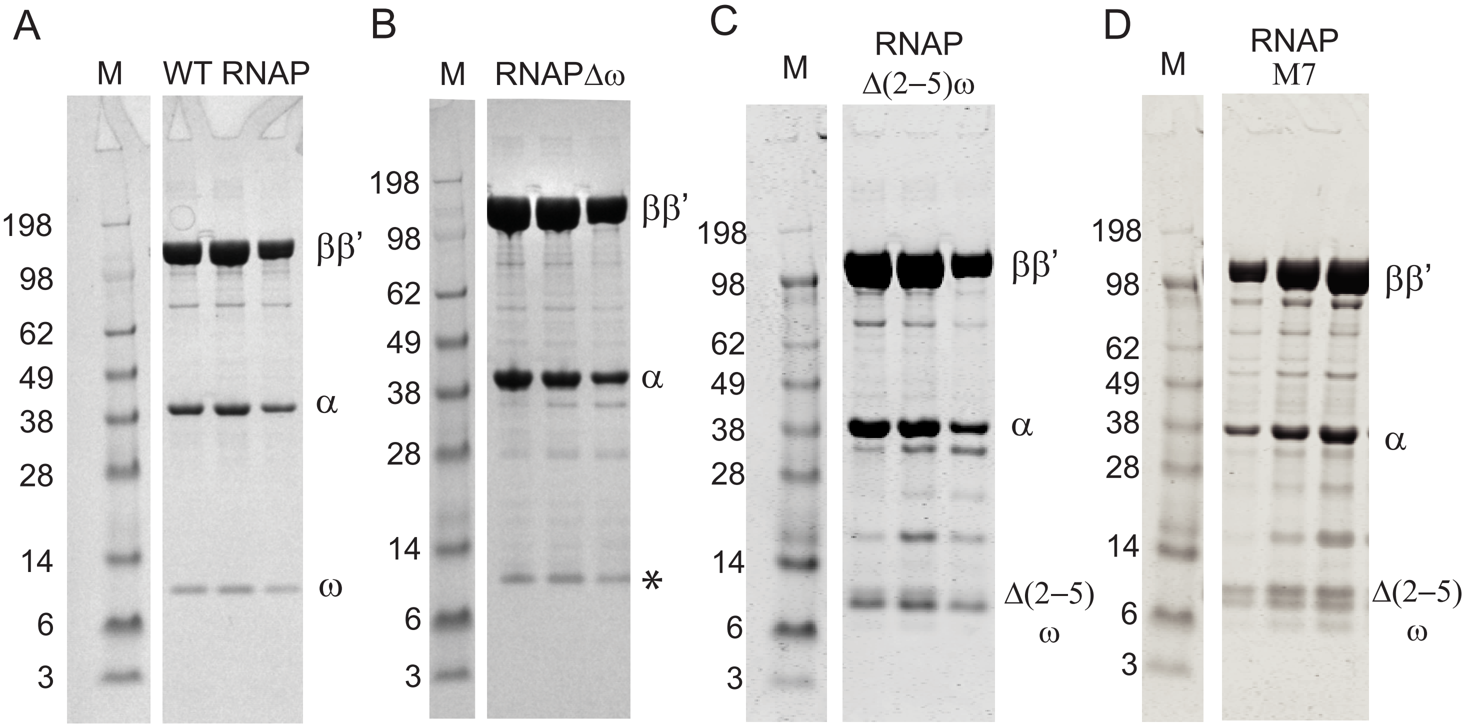

Figure S1. SDS-PAGE analysis of RNAP preparations. Peak fractions from the heparin sepharose chromatography step are shown for each RNAP. The size marker (M) was See Blue 2 (Invitrogen). Pooled fractions were dialyzed into DRaCALA buffer for ^{32}P-ppGpp binding experiments. **(A)** Wild-type RNAP was purified from strain RLG 15335 (BL21 DE3 *ΔdksA* with αββ'-His10 overexpression plasmid pRLG9960). (B) RNAPΔω was purified from strain RLG 14698 (BL21 DE3 *ΔrpoZ* with pRLG9960). Asterisk (*) marks a contaminant that does not react with anti-ω antibody (see Figure S2). **(C)** RNAP(Δ2-5)ω was purified from strain 14751 (BL21 DE3 *ΔrpoZ* pRLG9960 pCDFω(Δ2-5). **(D)** RNAP M7 was purified from RLG14293 (BL21 DE3 *rpoZ::kan,* pCDFωΔ2-5, pIA299 *rpoC*-His6 R362A, R417A, K615A) . This RNAP showed no ppGpp-dependent responses *in vitro* or *in vivo* (Ross, *et al.*, 2016; Sanchez-Vazquez et al., 2019).

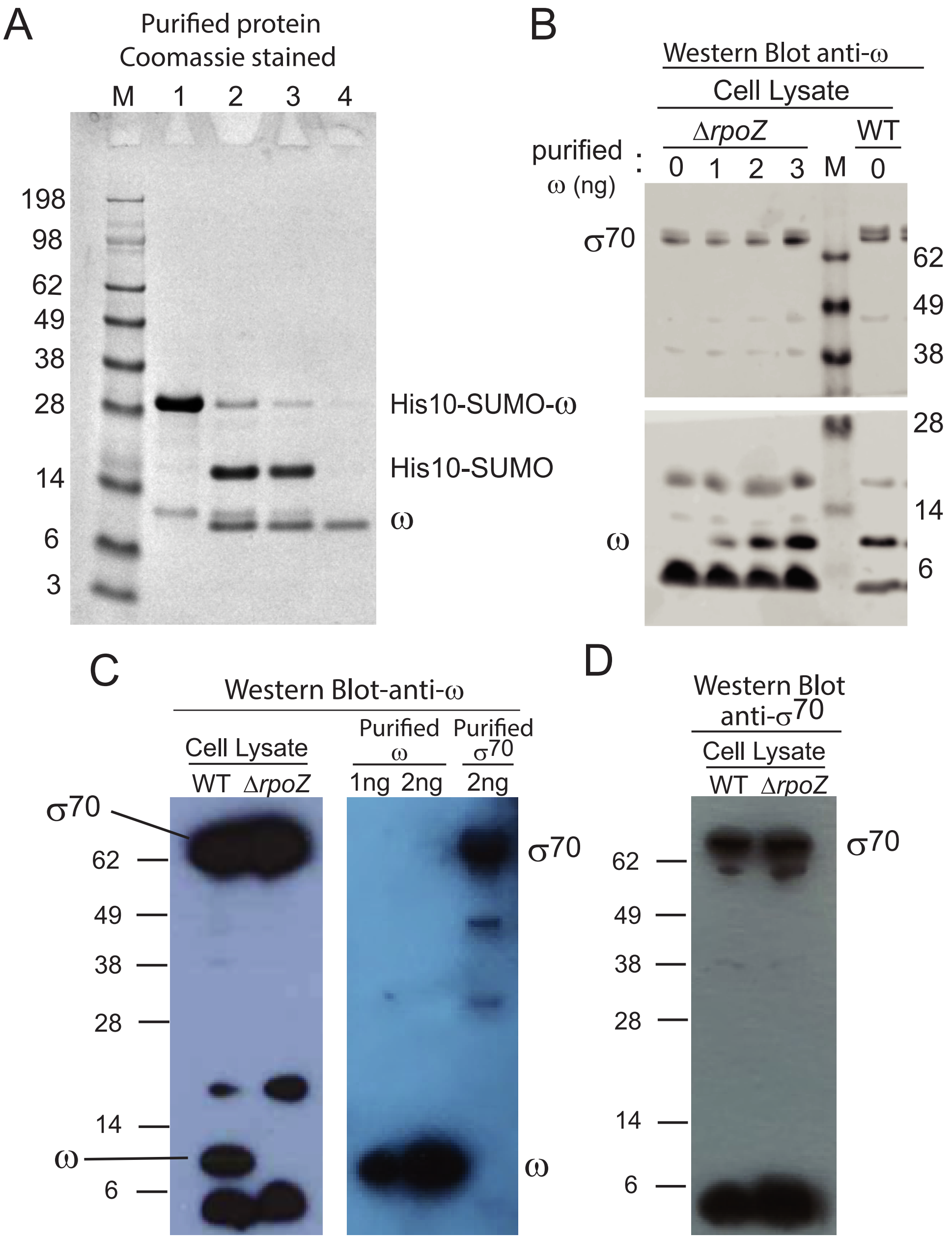

Figure S2. **Purification of ω and properties of anti-ω antiserum**. The size marker (M) in each panel was See Blue 2 (Invitrogen). **(A)** ω was purified using a SUMO tag as described in Extended Materials and Methods using pET23a-His10-SUMO-*rpoZ* (RLG15371). Lane 1: His10-SUMO-ω purified by Ni-NTA affinity chromatography. Lanes 2,3: Products of digestion of purified His10-SUMO-ω with Ulp1 protease. Lane 4: purified ω from flow through fraction after removal of His10-SUMO by Ni-NTA chromatography. **(B)** Western blot of lysates from Δ*rpoZ* cells with added amounts of purified ω, or of a lysate from WT cells without added purified omega using anti-ω antibody. The anti-ω antibody did not react with a band at the position expected for wild-type ω in the Δ*rpoZ* strain (left lane). **(C)** Left panel: Western blot with cell lysates from WT or Δ*rpoZ* strains, as in (B). Right panel: Western blot showing reactivity with purified ω and with purified σ^{70}. **(D)** Western blot with anti-σ^{70} antibody showing reactivity with σ^{70} but not with ω in lysates from WT or Δ*rpoZ* strains.

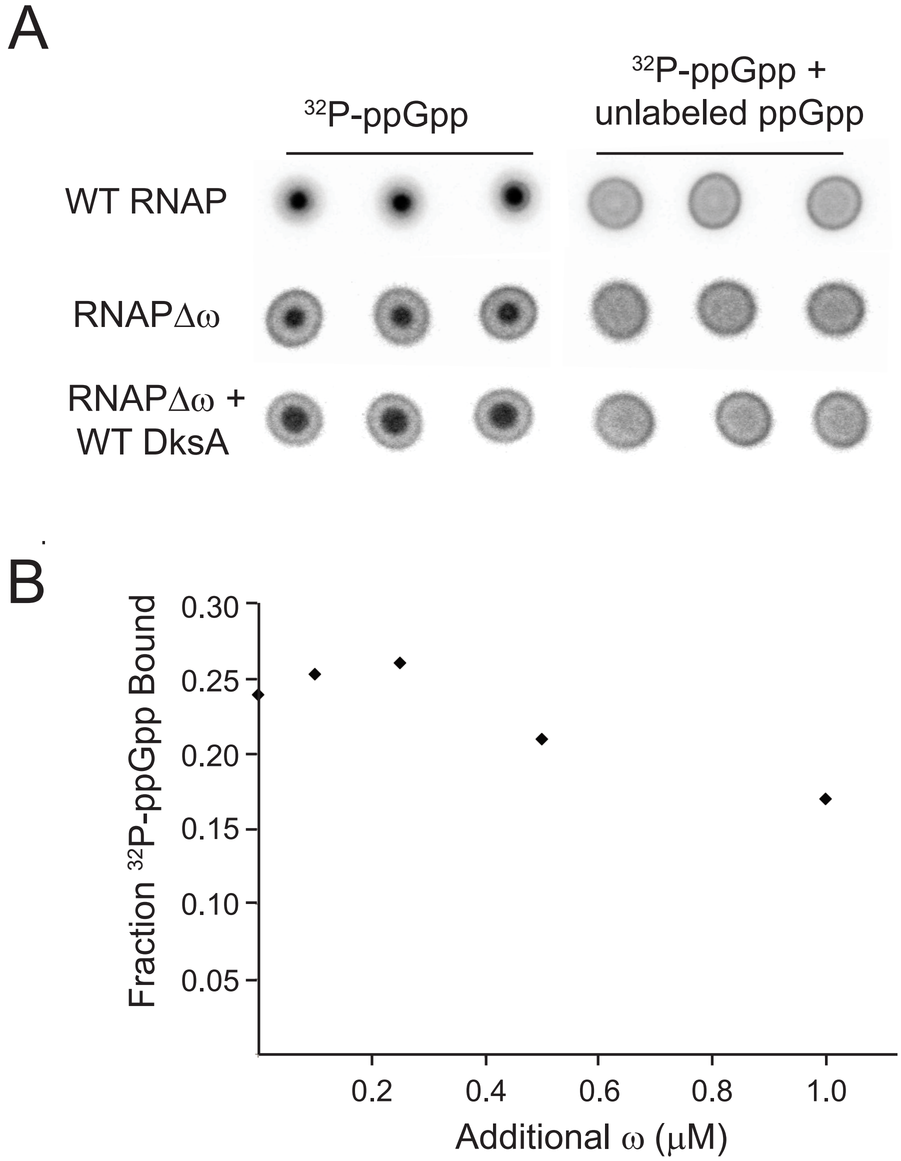

Figure S3. Specificity for ppGpp and ω saturation. (A) Unlabeled ppGpp competes with [^{32}P]-ppGpp for binding to Sites 1 and 2. Reactions containing 30 μM WT RNAP (top row), RNAPΔω (middle row), or RNAPΔω with DksA (bottom row) were equilibrated with [^{32}P]-ppGpp or [^{32}P]-ppGpp and 1 mM unlabeled ppGpp in DRaCALA buffer, then spotted onto nitrocellulose filters in triplicate. One representative experiment is shown for each condition. **(B)** Lack of effect of addition of indicated concentrations of purified ω to 0.5 μM wild-type RNAP (containing ω in the absence of DksA) on binding of [^{32}P]-ppGpp to Site 1 as determined by DRaCALA.

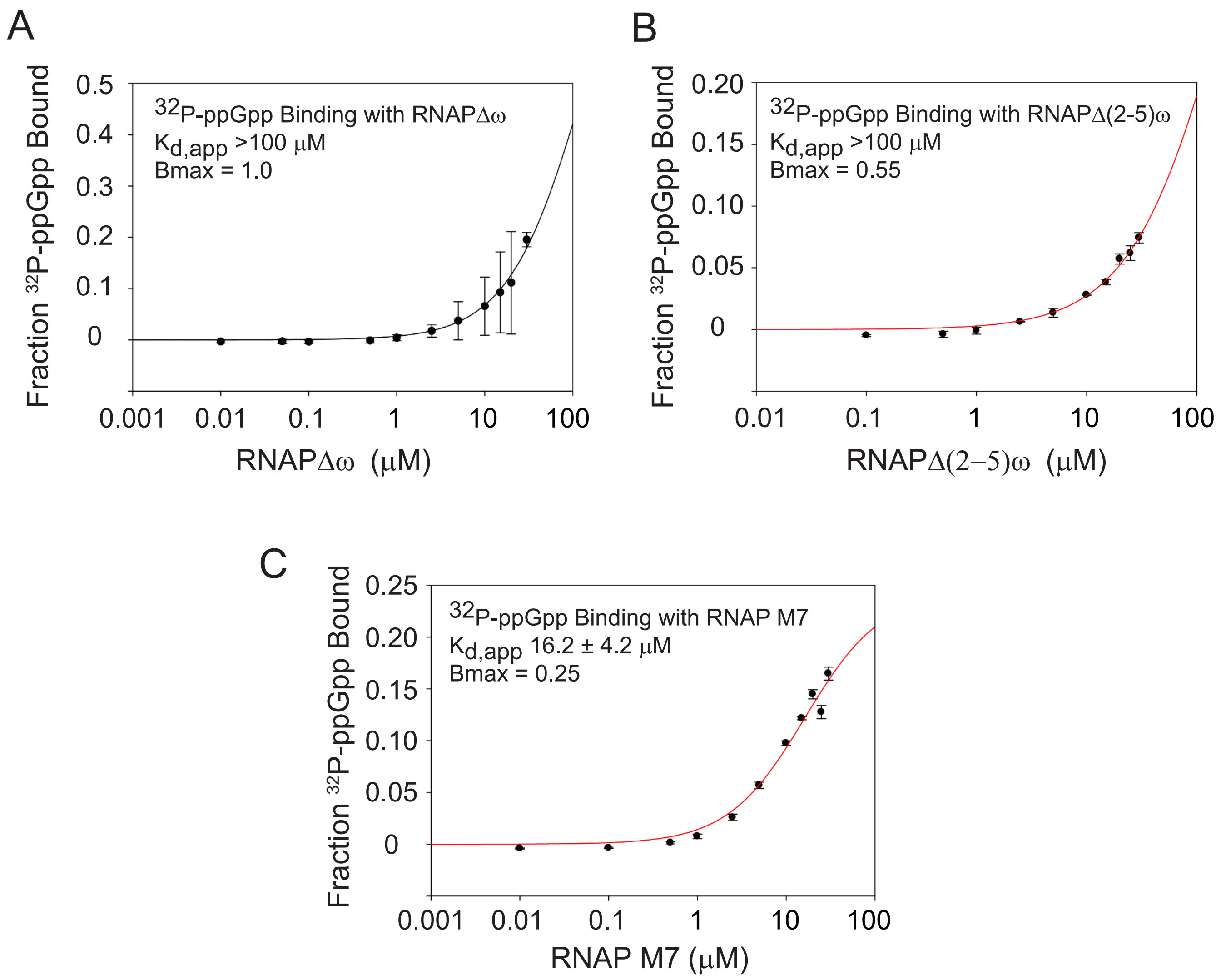

Figure S4. Binding affinities of [^{32}P]-ppGpp for RNAPs lacking functional ppGpp binding sites. The $K_{d,app}$ and B_{max} for each panel is indicated in the inset. The three panels correspond to RNAPs that derive from different mutations in *rpoZ* and/or *rpoC* eliminating Site 1 function. No DksA was added to the preparations, so each also lacked Site 2 (Ross et al., 2016). Increasing amounts of the RNAPs were equilibrated with ~5 nM [^{32}P]-ppGpp and assayed by DRaCALA. **(A)** The $K_{d,app}$ for [^{32}P]-ppGpp binding to RNAPΔω was determined from 7 separate experiments by fitting the data for each experiment to a one site binding curve and then showing the average [^{32}P]-ppGpp binding for all experiments. The error bars represent one standard deviation from the mean. The B_{max} parameter was set to 1.0 as none of the seven experiments reached saturation. **(B)** Same as (A) except the RNAP contained ωΔ(2-5). The fraction of [^{32}P]-ppGpp bound to RNAP ωΔ(2-5) was averaged from one experiment with filters in triplicate, fit to a one-site saturation ligand binding curve to determine $K_{d,app}$ and the B_{max}. Error bars represent one standard deviation from the mean. **(C)** Fraction of [^{32}P]-ppGpp bound to RNAP M7 averaged from triplicate filters from one experiment, as in (B). RNAP M7 contains ωΔ(2-5) and three additional substitutions in Site 1 that completely eliminate effects of ppGpp on transcription in the absence of DksA in vitro. It appears that these substitutions allow ppGpp to bind with only 2 to 3-fold weaker affinity than with wild-type RNAP, but in a manner that is non-functional (see text).

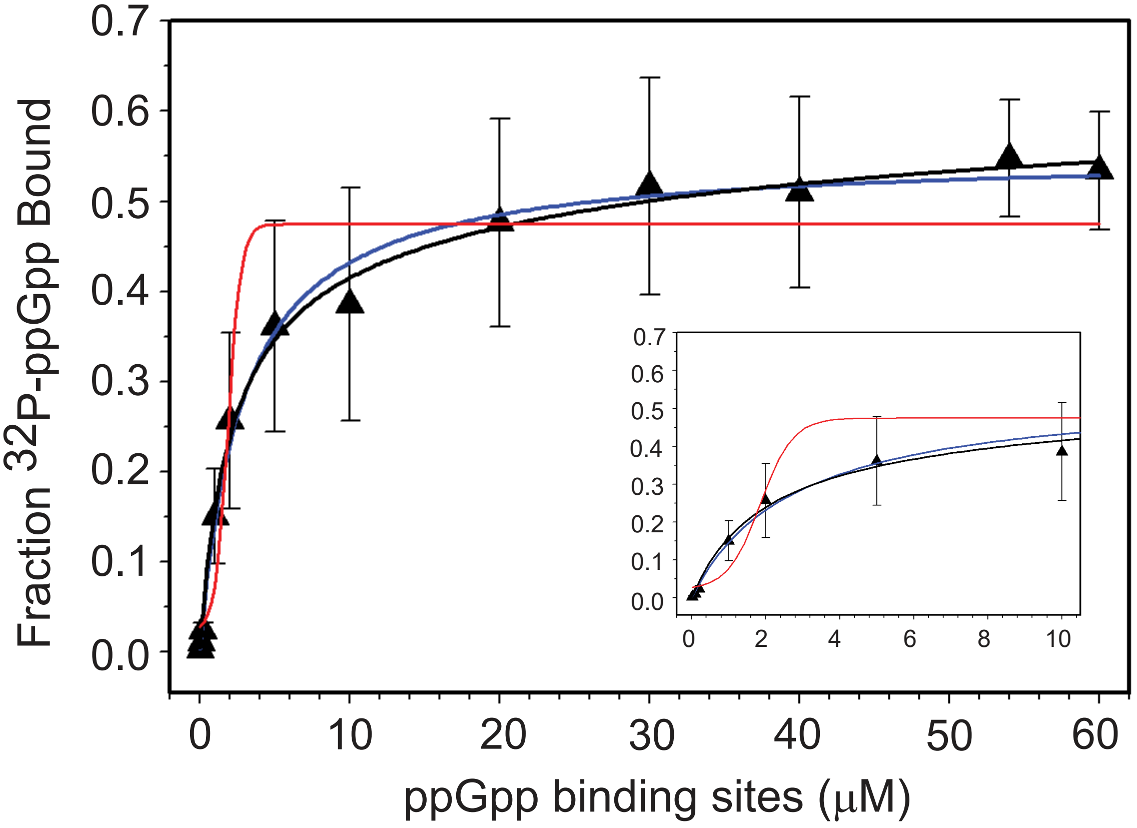

Figure S5. ppGpp binding to RNAP + DksA shown as a linear plot of fraction ^{32}P-ppGpp bound as a function of the concentration of ppGpp binding sites on RNAP (μM). The concentration of ppGpp sites indicated is twice the concentration of RNAP, since there are two ppGpp binding sites on RNAP. The data are the same as in the experiment shown in Figure 3, but they are shown here as a linear plot rather than a semi-log plot to evaluate whether binding to the two sites displayed cooperativity. The data were fit in SigmaPlot 10 to a ligand binding two site saturation equation ($y=(B_{max1}/K_{d1} + X)$ + $(B_{max2}X/K_{d2} + X)$; black line) or a ligand binding sigmoidal dose-response equation ($y=$ min $+[($max-min$)/1 + 10^{(\text{logEC50-x})\text{Hillslope}}]$); red line). A fit of the data to a ligand binding one site saturation equation ($y=B_{max}X/Kd +X$); blue line) is shown for comparison. Inset shows close up of low concentration range portion of the graph (0-10 μM ppGpp binding sites).

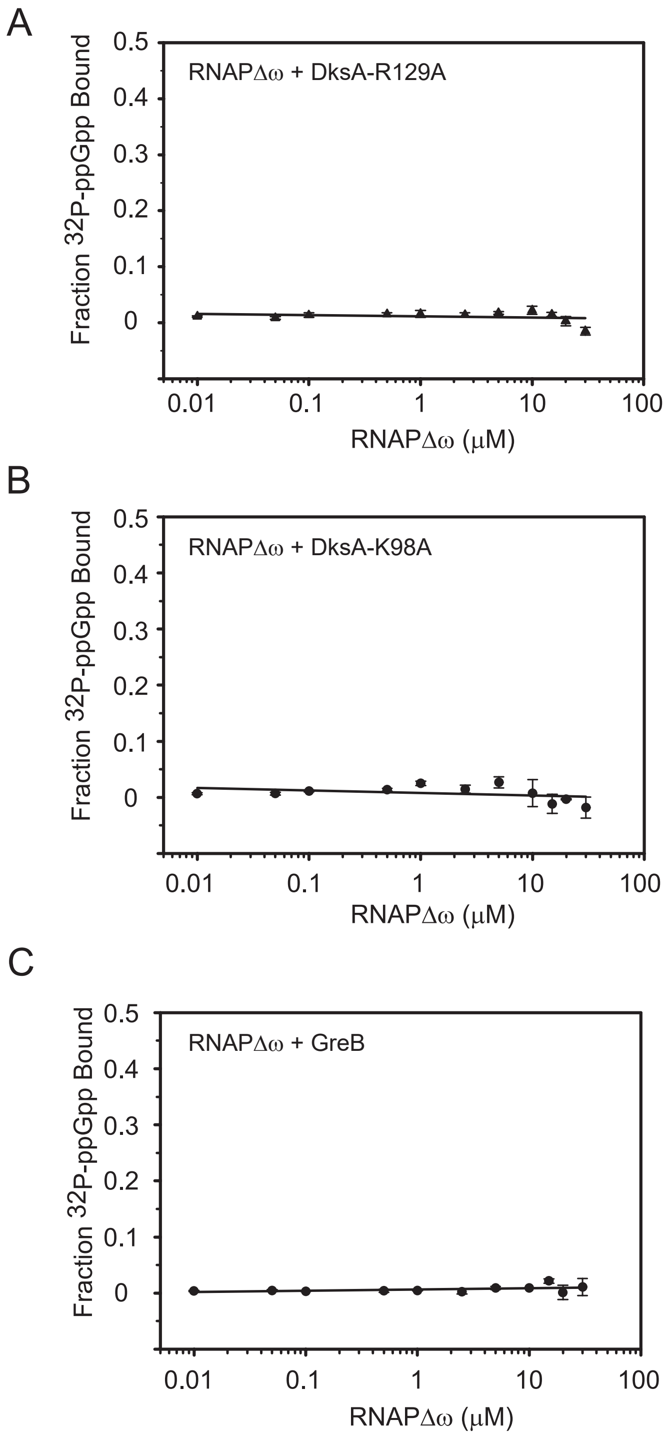

Figure S6. Binding of DksA variants or GreB to RNAPΔω does not result in binding of [^{32}P]-ppGpp to the complex. These results confirm that the mutant DksAs and GreB do not form a Site 2 like that formed by wild-type DksA. Increasing amounts of RNAPΔω (i.e. lacking Site 1) were incubated with **(A)** DksA-R129A, **(B)** DksA-K98A, or **(C)** GreB, equilibrated with ~ 5 nM [^{32}P]-ppGpp in DRaCALA buffer, and spotted onto nitrocellulose filters. Two separate experiments were performed in each case with filters in triplicate. Error bars represent one standard deviation from the mean. DksA-R129A and DksA-K98A are separation of function mutants, i.e. they bind to RNAP but not to ppGpp (Ross et al., 2016). We have shown previously that RNAPΔω-TraR complexes do not bind ppGpp (Gopalkrishnan et al., 2017)

Supplemental References

Baba, T., Ara, T., Hasegawa, M., Takai, Y., Okumura, Y., Baba, M., et al. (2006). Construction of *Escherichia coli* K-12 in-frame, single-gene knockout mutants: the Keio collection. *Mol. Syst. Biol.* 2:2006.0008. doi: 10.1038/msb4100050

Chen, J., Gopalkrishnan, S., Chiu, C., Chen, A.Y., Campbell, E.A., Gourse, R.L., et al. (2019). *E. coli* TraR allosterically regulates transcription initiation by altering RNA polymerase conformation. *Elife* 8:e49375. doi: 10.7554/eLife.49375

Gopalkrishnan, S., Ross, W., Chen, A.Y., and Gourse, R.L. (2017). TraR directly regulates transcription initiations by mimicking the combined effects of the global regulators DksA and ppGpp. *Proc. Natl. Acad. Sci. USA* 114, E5539-E5548. doi: 10.1073/pnas.1704105114

Henry, K.K., Ross, W., Myers, K.S., Lemmer, K.C., Vera, J.M., Landick, R., et al. (2020). A majority of *R. sphaeroides* promoters lack a crucial RNA polymerase recognition feature, enabling coordinated transcription activation. *Proc. Natl. Acad. Sci. USA*, in press.

Koulich, D., Orlova, M., Malhotra, A., Sali, A., Darst, S.A., and Borukhov, S. (1997). Domain organization of *Escherichia coli* transcript cleavage of GreA and GreB. *J. Biol. Chem.* 272, 7201-7210. doi: 10.1074/jbc.272.11.7201

Lee, J.-H., Lennon, C.W., Ross, W., and Gourse, R.L. (2012). Role of the coiled-coil tip of *Escherichia coli* DksA in promoter control. *J. Mol. Biol.* 416, 503–517. doi: 10.1016/j.jmb.2011.12.028

Paul, B.J., Barker, M.M., Ross, W., Schneider, D.A., Webb, C., Foster, J.W., et al. (2004). DksA: a critical component of the transcription initiation machinery that potentiates the regulation of rRNA promoters by ppGpp and the initiating NTP. *Cell* 118, 311–322. doi: 10.1016/j.cell.2004.07.009

Roelofs, K.G., Wang, J., Sintim, H.O., and Lee, V.T. (2011). Differential radial capillary action of ligand assay for high-through put detection of protein-metabolite interactions. *Proc. Natl. Acad. Sci. USA* 108, 15528-15533. doi: 10.1073/pnas.1018949108

Ross, W., Sanchez-Vazquez, P., Chen, A.Y., Lee, J.-H., Burgos, H.L., and Gourse, R.L. (2016). ppGpp binding to a site at the RNAP-DksA interface accounts for its dramatic effects on transcription initiation during the stringent response. *Mol. Cell* 62, 811-823. doi: 10.1016/j.molcel.2016.04.029

Ross, W., Vrentas, C.E., Sanchez-Vazquez, P., Gaal, T., and Gourse RL. (2013). The magic spot: a ppGpp binding site on *E. coli* RNA polymerase responsible for regulation of transcription initiation. *Mol. Cell.* 50, 420–429. doi: 10.1016/j.molcel.2013.03.021

Vrentas, C.E., Gaal, T., Berkmen, M.B., Rutherford, S.T., Haugen, S.P., Vassylyev, D.G., et al. (2008). Still looking for the magic spot: the crystallographically defined binding site for

ppGpp on RNA polymerase is unlikely to be responsible for rRNA transcription regulation. *J. Mol. Biol*. 377, 551–564. doi: 10.1016/j.jmb.2008.01.042

Vrentas, C.E., Gaal, T., Ross, W., Ebright, R.H., and Gourse, R.L. (2005). Response of RNA polymerase to ppGpp: requirement for the omega subunit and relief of this requirement by DksA. *Genes Dev*. 19, 2378–2387. doi: 10.1101/gad.1340305
